# Predicting the functional repertoire of an organism from unassembled RNA–seq data

**DOI:** 10.1186/1471-2164-15-1003

**Published:** 2014-11-20

**Authors:** Manuel Landesfeind, Peter Meinicke

**Affiliations:** Department of Bioinformatics, Institute for Microbiology and Genetics, Georg–August–University, Goldschmidtstraße 1, 37077 Göttingen, DE Germany

**Keywords:** Transcriptomics, RNA–seq, Metabolism, Reconstruction of metabolic pathways, Bioinformatics, Computational biology

## Abstract

**Background:**

The annotation of biomolecular functions is an essential step in the analysis of newly sequenced organisms. Usually, the functions are inferred from predicted genes on the genome using homology search techniques. A high quality genomic sequence is an important prerequisite which, however, is difficult to achieve for certain organisms, such as hybrids or organisms with a large genome. For functional analysis it is also possible to use a *de novo* transcriptome assembly but the computational requirements can be demanding. Up to now, it is unclear how much of the functional repertoire of an organism can be reliably predicted from unassembled RNA-seq short reads alone.

**Results:**

We have conducted a study to investigate to what degree it is possible to reconstruct the functional profile of an organism from unassembled transcriptome data. We simulated the *de novo* prediction of biomolecular functions for *Arabidopsis thaliana* using a comprehensive RNA-seq data set. We evaluated the prediction performance using several homology search methods in combination with different evidence measures. For the decision on the presence or absence of a particular function under noisy conditions we propose a statistical mixture model enabling unsupervised estimation of a detection threshold. Our results indicate that the prediction of the biomolecular functions from the KEGG database is possible with a high sensitivity up to 94 percent. In this setting, the application of the mixture model for automatic threshold calibration allowed the reduction of the falsely predicted functions down to 4 percent. Furthermore, we found that our statistical approach even outperforms the prediction from a *de novo* transcriptome assembly.

**Conclusion:**

The analysis of an organism’s transcriptome can provide a solid basis for the prediction of biomolecular functions. Using RNA-seq short reads directly, the functional profile of an organism can be reconstructed in a computationally efficient way to provide a draft annotation in cases where the classical genome-based approaches cannot be applied.

**Electronic supplementary material:**

The online version of this article (doi:10.1186/1471-2164-15-1003) contains supplementary material, which is available to authorized users.

## Background

The inference of gene functions remains essential in the analysis of *de novo* sequenced organisms granting first insights into the organisms metabolic potential. Reference databases such as the Kyoto Encyclopedia of Genes and Genomes (KEGG)[1, 2] and MetaCyc[3, 4] provide comprehensive information on metabolic pathways, biochemical reactions, and biomolecular functions for a large number of known organisms. In particular, the databases link gene– and protein–sequences with annotated functions. Based on these references, bioinformatic tools can be used to predict the functional repertoire of a newly sequenced organism. If a comprehensive list of gene functions is available then also the reconstruction of metabolic pathways[5, 6] is possible.

The functional capabilities of an organism are usually predicted from its genome[7]. Next–generation sequencing (NGS) technologies[8] allow the comprehensive sequencing with high coverage at low cost resulting in millions of short reads. These reads are merged into contigs and scaffolds using dedicated assembly tools[9–11]. Afterwards, all genes have to be identified on the contig sequences and the predicted genes are functionally annotated by homology search using tools such as BLAST[12]. For this functional annotation pipeline, a high quality genome sequence is required to achieve a sufficient prediction accuracy. While NGS assembly is still a challenging problem[13, 14], the genome-based approach is nevertheless well-suitable for most of the organisms investigated as of today. However, an increasing number of organisms are examined where genome sequencing and assembly is difficult to realize. Organisms with a huge genome[15] are hard to sequence with an acceptable genomic coverage. Further, hybrid organisms[16, 17] often originate from closely related species and make an assembly of short reads nearly impossible due to the high similarity between the duplicated chromosomes. Combinations of different sequencing and assembly strategies may still be able to cope with these problems, however, the cost may rapidly increase in such cases[18].

To circumvent the difficulties of the genome-based approach, pure transcriptomic analyses are a promising alternative to investigate these organisms. RNA–seq[19] allows the *de novo* sequencing of an organism’s transcriptome similar to genomic NGS approaches. Although the transcriptome in general only includes a subset of all gene functions encoded in the genome, RNA–seq data from different environmental conditions can show a broad spectrum of the metabolic capabilities. In particular, when using a suitable abstraction for the description of gene functions, orthology-based databases, such as KEGG[1, 2] or KOG[20] can yield a good overview of the functional repertoire.

In recent years, specialized tools have been developed for *de novo* transcriptome assembly without a reference genome[21, 22]. The assembled RNA contigs can then be used to predict the organisms functional profile directly, using the same techniques as in the genome-based approach. Just like genomic assembly, the *de novo* assembly of RNA–seq data requires high end computer hardware to process the enormous amount of reads. Thus, for many researchers it would be a highly desirable option to infer the functional profile of an organism directly from unassembled RNA–seq short read data.

Although the same homology search tools may be applied to assign RNA–seq short reads to protein families with annotated functions, the short length of sequencing reads may severely affect the quality of functional predictions. Because short reads would frequently show similarity to multiple protein families that share conserved domains, the mapping to functions is inherently ambiguous. Because this ambiguity cannot be resolved in principle, the commonly used best-hit assignment easily results in many spurious predictions.

In contrast to a prediction on an assembled contig, a single short read assignment to a function generally does not provide enough evidence for the presence of that function. Hence, the abundance and the quality of read assignments to functional categories have to be taken into account to increase the evidence for a more reliable prediction. To rule out false predictions, functions have to be filtered according to some suitable evidence threshold.

To investigate the usability of RNA–seq short read data for the prediction of the functional capabilities of an organism, we conducted a case study using a publicly available transcriptome dataset of *A. thaliana* from the NCBI database. The short reads are mapped to functionally classified amino acid sequences from the KEGG database using four state–of–the–art homology search tools. Based on their best–hit, the short reads are assigned to functions and evidence values are estimated for all functions. These evidence values are further analyzed using an unsupervised mixture model approach to determine an optimal prediction threshold for filtering of false positives. Finally, the results from all homology search tools in combination with two different evidence estimation methods are compared utilizing common performance measures. In this study, we were able to reconstruct the functional repertoire of *A. thaliana* with a sensitivity of up to 94 percent and a specificity above 95 percent. Our results indicate that unassembled RNA–seq short reads can directly be used to predict the functional capabilities of an organism with a high confidence. Thus, pathway reconstruction can be performed efficiently even in situations where the classical genome-based approach is not feasible. Further, we applied our short read approach to the transcriptome of a fungi to demonstrate the versatility of the method.

## Methods

Our method for predicting the presence or absence of a particular function is based on the discrimination between strong and weak sequence homologies based on different degrees of similarity with respect to protein families from a broad range of organisms. This discrimination is realized by a statistical mixture model for analyzing the evidence measure as derived from the frequencies and similarity scores of short read assignments.

In our study we utilized the model plant *A. thaliana* as a test organism to evaluate the performance of the prediction of functions from unassembled RNA short reads based on existing annotations in KEGG. In the following, we outline the study setup, utilized data, and methods. A schema of the implemented work–flow is shown in Additional file1.

### Database of functionally classified proteins

In our study, functions are represented by KEGG Ortholog groups (KO). Here, all potential functions are represented by specific sets of amino acid sequences from different domains of life including bacteria, plants, and animals. The RNA–seq reads are compared against these protein families where similarity hits yield some evidence for the corresponding function.

From the KEGG database, we downloaded all amino acid sequences that are annotated with a KO^a^ and combined them into a multiple FASTA file. Sequences originating from *Brassicaceae*^b^ were removed to simulate the analysis of a "novel" organism that has no close relatives included in the database. The final FASTA file contained more than 4.87 million sequences from 16,423 KOs associated with 2881 organisms.

### Assignment of RNA–seq reads to functions

We utilize four tools to assign the RNA–seq reads to the KO protein sequences. The standard tool for homology search is BLAST ([12], version NCBI BLAST 2.2.26+), in our context BLASTX, because DNA sequences are aligned to proteins sequences. BLASTX is computationally expensive and therefore only 10 percent of each sample are processed. The RAPSearch ([23], version 2.12) and PAUDA[24] tools accelerate BLASTX by using a reduced amino acid alphabet of 10 different symbols for RAPSearch and four for PAUDA ([25], version 1.0.1). Thereby, RAPSearch and PAUDA are about two and four magnitudes faster than BLASTX, respectively. Finally, we use the Ultrafast Protein Classification tool ([26], version 1.1.1) which implements a direct classification of the reads to KOs based on inexact word matches and machine learning.

Specific databases for all tools were generated by the corresponding software based on the KO FASTA file (see section Database of functionally classified proteins). For the homology search, we set very liberal thresholds: the BLASTX and RAPSearch E–value cut–off is set to 10 and the protein threshold of Uproc to 0. Unfortunately, PAUDA internally uses fixed thresholds for filtering but does not offer an option to change them. Therefore, we employed PAUDA using its standard cut–off which results in a low number of read assignments (see Results). Additionally, we activate the "short read" option of Uproc. The result of each tool contains mappings of the reads to KOs and report the quality of the mapping in terms of a score, e.g. the bit score from BLASTX. For BLASTX, RAPSearch, and PAUDA the mapping can be ambiguous for a single read. This ambiguity is resolved by considering only the hit with the highest score ("best hit").

### Estimation of evidence for metabolic functions

Using the KEGG database for functional annotation, a function is considered to be present in an organism if there exists a high similarity between a protein of the organism under investigation and a functionally annotated protein in the reference database of KEGG orthologs. Because sequence homology may also exist with respect to distant organisms, for example plant protein sequences often show similarities to bacterial protein families, the similarity–based prediction of functions must be able to distinguish between strong and weak homologies. For that reason we employ an adaptive two-component mixture model for analysis of the sequence–based evidence measure. We calculate an evidence measure for each function *f* based on the scores of the assigned hits *H*_*f*_. In this study, we evaluate four distinct evidence measures:

-  *count* refers to the number of hits for a particular function
-  *sum–of–scores* sums up the scores of all hits to a function
-  *mean–score* is the average hit score
-  *scaled mean–score* (*SMS*) adjusts the *mean–score* with the ratio of the log–scaled counts to the maximum counts

The *count* and *sum–of–scores* measures are biased by gene expression level but commonly used because they are easy to interpret. Therefore, we also consider the above mean–based measures that are less influenced by the expression level.

### Quality filtering using automatic threshold estimation

Because we utilize liberal thresholds for homology search of the RNA–seq short reads, also weak hits contribute their score to the evidence measures of the functions. Therefore, some evidence is found for most of the functions in the database and a statistical mixture model is used to discriminate between strong ("true") and weak ("false") evidence.

For each sample all possible combinations of a particular tool and evidence measure are evaluated and an optimal threshold is determined employing a two-component mixture model. The models consist of two probability distributions that represent functions with low and high evidence, respectively. After fitting the model to the observed evidence values, the optimal threshold is determined by minimizing the risk for false predictions. Functions with a high evidence value above that threshold are considered present in a particular sample for the corresponding tool and measure combination.

For the *mean–score* measure, a mixture of two Gaussian distributions is employed which is fitted by an EM algorithm[27]. The *scaled mean–score* values are modeled by two Gamma distributions that are fitted by the SEM algorithm[28, 29].

### Using replicates for consensus prediction

The five technical replicates (see Section Transcriptomic RNA–seq dataset) are processed independently with regard to each tool and evidence measure. Because the short read data varies in different replicates, a function will gain different evidence values and might be filtered differently. We evaluate the robustness of our approach in terms of the stability of results for a specific tool and measure combination by comparing the prediction performances across replicates. Additionally, we test a consensus–based approach to further reduce the number of false positive predictions. Hereby, a function is considered present if it is predicted in a minimum number *c* of replicates. Note that the consensus approach is expected to remove false positives but may also eliminate true functions. Therefore, the prediction quality is evaluated for different consensus thresholds ranging from one to five replicates for the *A. thaliana* data set.

### Transcriptomic RNA–seq dataset

In contrast to the genome, the transcriptome of an organism varies with respect to the environment. For prediction of the complete range of biomolecular functions, RNA–seq data is required that covers a broad variety of environmental conditions. For higher eukaryotes, also a variety of different tissues and growth stages has to be sampled.

For the case study, RNA–seq data of *A. thaliana Col-0* is utilized originating from an experiment that investigates alternatively spliced genes[30, 31]. The RNA of flowers and 10–day seedlings was pooled and from this pool five technical replicates were sampled and sequenced. The samples consist of approximately 10 to 30 million single–end reads of about 150 bp length resulting in a total of about 110 million reads. We used PRINSEQ[32] to filter out reads with an average Phred score lower than 25. Each of the five samples was processed independently.

Using Bowtie2[33] we mapped the reads to the 7489 gene sequences of *A. thaliana* that are annotated with a KO^c^. From each sample at least 35% of the reads hit at least one of the genes. In total, mappings were found to 7311 genes (98%) covering 3141 annotated functions (99.9%). Therefore, the dataset is actually suitable to characterize the full functional repertoire in terms of the KEGG Orthologs.

### Evaluation of prediction performance

The utilized KEGG database annotates 3,146 functions (KOs) for *A. thaliana* that we consider as positives (P) while all other functions are negatives (N). In our evaluation, we count predicted and annotated KOs as true positives (TP) while false positives (FP) correspond to predicted but not annotated functions. We then measure the prediction accuracy in terms of the true positive rate (or sensitivity:), the false positive rate, the specificity (1- false positive rate), and positive predictive value. The F1–Score, the harmonic mean of precision and sensitivity, is used as single index for the prediction quality after filtering.

### *De novo*transcriptome assembly

Additionally, we compare our novel method to the predictions obtained via *de novo* transcriptome assembly. We assembled the RNA contigs of each sample using IDBA-tran[22] and utilized BLASTX to search for homologous sequences in the same database that was used for the short read assignment. The performance of this approach is evaluated for different E–value thresholds ranging from 10 to 10^-100^.

## Results

In this study, we predicted the functional repertoire of *A. thaliana* from RNA–seq data by assignment of short reads to biomolecular functions in terms of KEGG Orthologs (KO) using four homology search tools. All software tools were able to assign large numbers of reads to the KOs from the reference database (see Additional file2). Only PAUDA mapped a smaller amount of reads because it internally uses restrictive thresholds. Except for PAUDA, all tools also hit a large number of KOs.

Regarding runtime there were large differences^d^: UProC required about 10 to 15 minutes per sample depending on the actual size, PAUDA took 30 to 60 minutes, while RAPSearch needed 35 to 40 hours. BLASTX required several days to process 10 percent of a sample which led to an estimated runtime of 3 to 5 weeks per complete sample.

### Evaluation of scoring methods

In the estimation of the evidence for each function we compared four distinct measures (values are provided in Additional file3). The performance of the different evidence measures was evaluated first to determine their general suitability as a measure for prediction. Receiver Operating Characteristic (ROC) and Area–Under–Curve (AUC) for the different tools and evidence measures (see Table1 and Additional file4) clearly showed that, on average, the functions annotated for *A. thaliana* gain higher evidence than other functions. The AUC was above 0.92 for all methods and tools (Table1). Tool–wise comparison of the AUCs showed that *mean–score* and *scaled meanscore* are more suitable than *count* and *sum–of–scores* evidence measures. We additionally evaluated the discriminative power of the different measures by means of the maximum achievable F1–Score (see Table1). Again, the mean–based evidence measures were superior. Therefore, we restricted our further evaluation to these measures.

**Table 1:** **Prediction performance for different tools before filtering**

Tool	Sensitivity	AUC (in %)			
	(in %)	***count***	***sum–of–***	***means–***	***scaled mean–***
			***scores***	***core***	***score***
BLASTX	97.21	92.02	93.09	**94.96**	94.95
RAPSearch	97.04	96.13	96.26	95.38	**96.78**
PAUDA	96.36	96.89	96.94	94.66	**97.07**
UProC	97.10	92.20	95.62	95.68	**96.03**
		**Maximum F1–Score (in %)**			
		***count***	***sum–of–***	***mean–***	***scaled mean–***
			***scores***	***score***	***score***
BLASTX		74.19	76.77	**84.60**	82.80
RAPSearch		87.03	87.72	86.13	**89.50**
PAUDA		88.30	88.63	83.09	**89.28**
UProC		75.01	87.67	88.60	**89.93**

The area under the curve (AUC) was calculated on sorted functions. The maximum F1–Score corresponds to the best possible separation between false and true predictions. Quality scores are averaged over all samples. The maximum AUC and F1–Score per tool are marked in bold text.

### Threshold calibration

**Figure 1:**
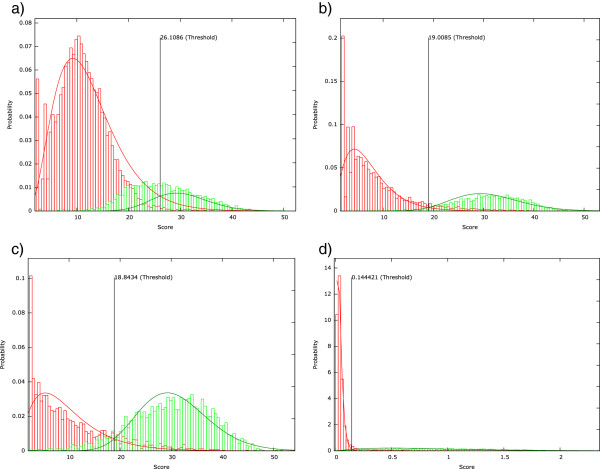
**Score distribution and fitted Gamma mixture model.** Histogram of scores from sample SRR360152 with threshold estimator using *scaled mean–score* and Gamma Mixture Model. The evidence value histograms of the falsely predicted and the annotated functions are colored in red and green, respectively. The curves correspond to the probability distributions of the two component mixture model. Although the probability density curves are shown colored in the plot, the fitting of the model was performed in an unsupervised manner. Histograms were generated from sample SRR360152 based on the results from BLASTX **(a)**, RAPSearch **(b)**, PAUDA **(c)**, and UProC **(d)**.

For each replicate sample and particular homology search tool, the evidence values were modeled by probability distributions as described in Section "Quality filtering using automatic threshold estimation". Histograms of the evidence values with fitted distributions (see Figure1 and Additional file5) showed that the fitting of the *mean–score* values for RAPSearch was problematic because the Gaussian distribution was not suitable to correctly model the falsely predicted functions. Therefore, we expected a large number of falsely predicted functions for this combination. Further, the fitting of the *scaled mean–score* values did not work well for BLASTX assignments because the evidence distributions for false and true functions are strongly overlapping which resulted in filtering out large numbers of annotated functions. Nonetheless, we calculated optimal filter thresholds from all models.

After application of the thresholds, we evaluated the prediction performance (see Table2, Additional files6 and7). As introduced in Table2, the filtered *MS* results are very sensitive but show a large number of false positives. As a results of the poor threshold calibration, 42% of the functions predicted from RAPSearch assignments are false positives. Compared to the *MS* evidence measure, filtered *SMS* results are a little less sensitive for UProC and RAPSearch but the number of false positive predictions is substantially lower. The combination of BLASTX with SMS measure is unsuitable because of the low sensitivity that originates from the overlapping evidence distributions.

**Table 2:** **Average performance after filtering**

***MS***	TPR	FPR	PPV	F1
BLASTX	92.85	8.57	72.64	81.50
RAPSearch	96.66	17.19	57.98	72.47
PAUDA	94.68	9.38	71.41	81.34
UProC	94.97	9.34	71.34	81.47
***SMS***	**TPR**	**FPR**	**PPV**	**F1**
BLASTX	54.94	1.00	93.60	68.54
RAPSearch	93.75	4.26	84.35	88.80
PAUDA	87.31	2.33	90.20	88.72
UProC	94.02	4.76	82.88	88.10

Performance averaged over all samples after filtering the *mean–score* (*MS*) using Gaussian mixture model and the *scaled meanŰscore (SMS)* by Gamma mixture model True positive rate (TPR), false positive rate (FPR), positive predictive value (PPV), and F1–Score (F1) are utilized as performance measures. All values are given in percent.

In terms of the F1–Score as a single performance index, the *SMS* evidence measure is superior to the *MS* measure for RAPSearch, PAUDA, and UProC. Comparing the achieved average F1–Scores with the maximal achievable F1–Scores (see Table1) displays that the filtering of the evidence values is close to optimal filtering in most cases.

### Consensus prediction evaluation

**Figure 2:**
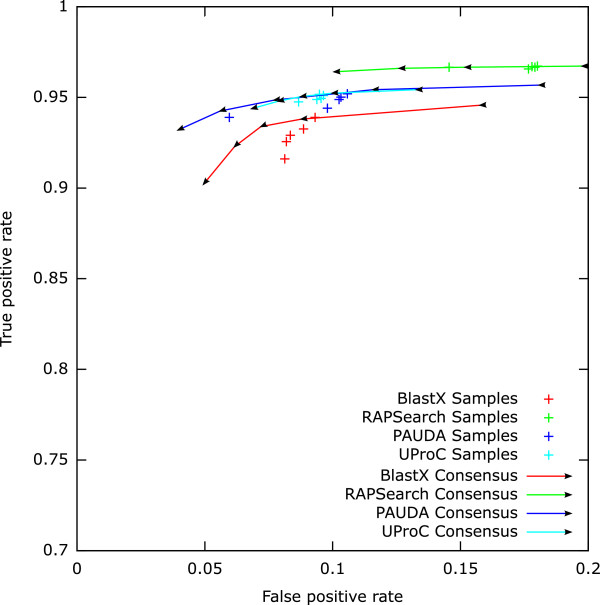
**Prediction performance after filtering and consensus on** ***mean–score*** **evidence values.** The arrows indicate the increasing consensus threshold ranging from one to five.

**Figure 3:**
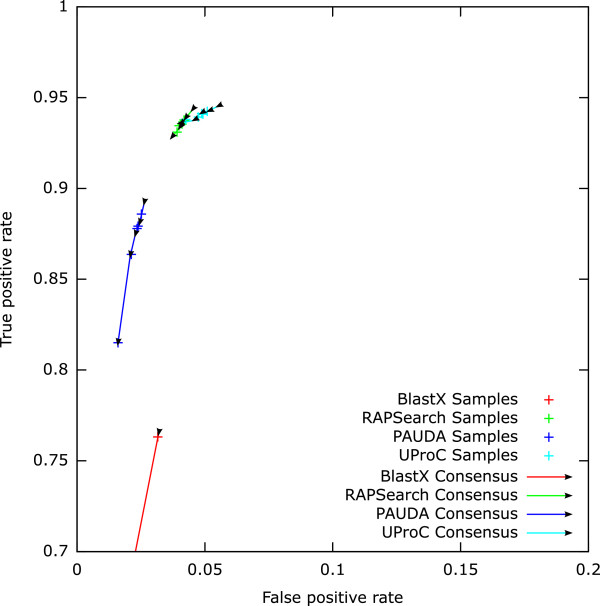
**Prediction performance after filtering and consensus on** ***scaled mean–score*** **evidence values.** The arrows indicate the increasing consensus threshold ranging from one to five.

We evaluated the robustness of our approach by means of the variation in prediction performance across the five samples. Results obtained via the *mean–score* (*MS*) measure show a high variation for all tools (see Figure2). Especially PAUDA and RAPSearch showed a single outlier. Also, the *scaled mean–score* (*SMS*) results based on BLASTX and PAUDA show a high variation while the *SMS* results from RAPSearch and UProC assignments were very robust (see Figure3).

To reduce the number of false positive predictions, we combined the five samples per tool by the consensus approach considering a function to be present if predicted in a given number of samples. The false and true positive rates for the *MS* results (see Figure2 and Additional file8) showed that an increasing consensus threshold considerably reduces the number of false predictions for all tools. However, results on RAPSearch assignments still contain large numbers of false positives for all thresholds as an effect of the inefficient filter threshold. PAUDA with *MS* evidence measure profits most from a consensus prediction. But for the *SMS* measure, the consensus prediction decreased sensitivity for PAUDA severely (see Figure3 and Additional file8). Again, BLASTX results with *SMS* measure are very insensitive as a result of the poor threshold calibration. For *SMS* based predictions from RAPSearch and UProC assignments, the consensus prediction had only slight influence on the performance.

The results suggest a preference for the UProC tool with the *scaled mean–score* evidence measure. While results from UProC and RAPSearch read assignments yielded a high prediction quality and robustness, UProC was much faster compared to RAPSearch. PAUDA was also able to achieve a high F1–Score using the *MS* evidence measure and a high consensus threshold but the single sample predictions were less robust.

### Comparison to *de novo*transcriptome assembly

To compare our method with existing approaches, we predicted the functional repertoire of *A. thaliana* using a *de novo* transcriptome assembly and BLASTX homology search with different E–value thresholds (see Table3). Here, we were also able to detect up to 95.6% of the functions annotated for *A. thaliana* using a high threshold of 10 for maximum sensitivity. Thereby, the assembly–based approach was only slightly more sensitive than our short read approach. Lower E–value thresholds increased specificity, but thresholds below 10^-50^ severely reduced sensitivity. For comparison, we examined the F1–Scores at different E–value thresholds in contrast to the F1–Scores achieved by our approach. Tables2 and3 clearly display, that our approach utilizing UProC read assignments and the *scaled mean–score* evidence measure performed better than the *de novo* assembly approach over the whole range of E–value thresholds. In terms of the F1–Score, the performance of our approach is at least 7% higher.

**Table 3:** **Performance of the functional prediction from** ***de novo*** **transcriptomic assembly using different E–value thresholds**

E-value cutoff	FPR	TPR	PPV	F1
10	89.63	95.69	20.16	33.30
1e-1	50.86	95.69	30.79	46.59
1e-5	35.67	95.59	38.79	55.19
1e-10	26.62	95.49	45.89	61.99
1e-25	15.24	94.31	59.41	72.90
1e-50	08.33	89.44	71.74	79.62
1e-75	04.59	80.43	80.56	80.49
1e-100	02.95	72.52	85.31	78.40

True positive rate (TPR), false positive rate (FPR), positive predictive value (PPV), and F1–Score (F1) are utilized as performance measures. All values are given in percent.

### Application

To demonstrate its versatility, we applied our novel method to the fungi *Verticillium dahliae JR2*[34]. The transcriptomics data set[35] only comprises four samples. The samples involve two different experimental conditions with two samples taken from a mutant. For the read assignments, we utilized UProC and calculated the evidence values with the scaled mean–score measure.

At first, we utilized the full KEGG database containing all 4.88 million protein sequences annotated with a KO. From the four samples, we predicted 2753 functions using a consensus threshold of three (see Additional file9). Although *V. dahliae JR2* was not contained in the KEGG database, we identified the closely related organism *V. alfalfae*. Therefore we repeated the experiment after removing *V. alfalfae* from the database. Without *V. alfalfae*, 2807 functions were predicted.

We compared these results with a prediction based on the proteome^e^ that predicted 2977 functions (see Additional file10). Using these functions as reference, our predictions with and without *V. alfalfae* proteins in the database achieved an F1–Score of 89% and 88%, respectively.

In both cases, we were able to efficiently map large amounts of the reads to the reference database and to calculate evidence values per function. The Gamma mixture models reflected the bimodality of the evidence distributions and therefore were useful to automatically determine prediction thresholds.

## Discussion

We investigated the potential of unassembled RNA–seq data for the prediction of the functional repertoire of an organism based on a statistical mixture model for discriminating between strong and weak sequence homologies.

Although we used *A. thaliana* as a test species for evaluation, our approach is not restricted or specialized on plants. The proposed method may in fact be used for a broad range of organisms which is also indicated in our application to the fungi *V. dahliae JR2*. The method should be most beneficial in situations where the genome of the organism under study or a closely related genome is not available. Therefore, our statistical approach is focused on particular applications and is not intended to provide a general tool for the improvement of functional annotation. In cases where the genome of the organism is sequenced and a well–annotated closely related genome exists, it is unlikely that our method can improve upon the classical annotation approach based on the identification of all orthologous genes. Further, it will generally not be possible with short read data alone to distinguish between orthologs and paralogs. This shortcoming clearly shows the limits of a merely homology–based inference. To finally overcome these limitations, the complete genome sequence and related organisms with high quality genome annotations would be required. Therefore, the main potential of our approach is to provide a draft annotation of the functional repertoire in cases where the classical genome-based approaches cannot be applied. This also suggests the application of our approach to metagenomic data where the clustering of sequencing reads[36–38] could be used as a basis to reconstruct the functional repertoire for the most abundant organisms in a microbial community.

A key element of our approach is the similarity–based assignment of RNA–seq short reads to functionally annotated protein families. In our study we evaluated and compared different software tools for read assignment that vary in methodology, speed, and general sensitivity. A particular requirement for our approach is the ability of the assignment tools to yield an evidence measure that is able to differentiate between weak and strong sequence similarities. For successful fitting of the evidence values by the two–component mixture model, also a probability distribution is required that is able to represent the variation of the observed values. Our case study was intended to identify combinations that can achieve a successful discrimination. Nonetheless, some of the results may be improved by more complex distribution models that can better represent the statistical variation of a particular evidence measure. For instance, the RAPSearch-based *mean–score* values might be modeled using a mixture of generalized extreme value probability distributions[39]. Based on our results so far, we would suggest the use of UProC in combination with the *scaled mean–score* measure because of computational speed and because the predictions show a good F1–Score while providing a high stability across replicates.

Finally, we would like to point out that our discriminative approach to the prediction of functions requires an orthology database that covers a broad range of organisms. Although such databases, like KEGG, may not reach the level of detail and the annotation quality of a more specialized resource, these databases are valuable for broad comparisons and widely used by biological researchers. In our case, it is essential that the spectrum of evidence values shows a bimodality that allows the discrimination between true and false candidate functions. Although, for all organisms that we analyzed so far we observed a strong bimodality, we cannot exclude that for organisms that are too far from any of the reference species in KEGG, the bimodality may become too weak for a reliable prediction. However, it is unlikely that in this case a more specialized database of closely related organisms is available for a better prediction.

## Conclusion

Our results show that it is possible to predict the functional repertoire of an organism based on unassembled RNA–seq high–throughput data. Compared to the classical genome–based approach, the use of RNA–seq short reads can substantially reduce the experimental effort and at the same time facilitates computation because there is no need for assembly and gene prediction. Furthermore, our approach extends the range of organisms that can be studied and can, for instance, be applied to organisms with complex genomes that complicate sequencing and assembly. The proposed method provides an important alternative when classical approaches are not applicable. In our evaluation of different homology search tools we found that UProC can achieve an ultra–fast assignment of reads to functions without loss of sensitivity compared to computationally more expensive tools. As a central feature of our approach, the proposed evidence measures in combination with a statistical mixture model enable an automatic calibration of the prediction threshold. Combining the UProC read assignment with the *scaled mean–score* evidence measure furthermore yields the most stable predictions across different replicate samples. In our KEGG-based evaluation of the prediction performance, about 94% of the annotated functions could be predicted with only 4% false positives. In this evaluation we also found that our short read approach achieved a better F1 performance than a computationally more expensive *de novo* transcriptome assembly approach.

## Endnotes

^a^KEGG FTP Release 2014-03-17.

^b^namely *A. thaliana*, *Arabidopsis lyrate*, *Capsella rubella*, and *Eutrema salsugineum*.

^c^Bowtie2 was applied with default options and –local mode.

^d^All homology search tools were executed on a compute server with Intel(R) Xeon(R) E7 CPUs (2 GHz) with 8 parallel threads. To annihilate influences of reading and writing files to the hard disk, all data was kept in memory.

^e^BLASTP homology search with e–Value threshold 10^-25^.

## Electronic supplementary material

Additional file 1:**Work flow of the study.** Visualization of the work-flow for this study. (PDF 130 KB)

Additional file 2:**Mapping statistics.** For each tool and sample, the number of mapped reads, hits, and distinct functions are given. PAUDA only maps approximately 17% of the query reads to the amino acid sequences in the database while BLASTX and RAPSearch both map about 40% of them. Most reads were classified by UProC. Because no stringent threshold is used for the mapping, all tools hit many false functions (about 3000 true functions are expected). Therefore, a filtering for false positive hits is required. (PDF 30 KB)

Additional file 3:**Evidence values.** These tables contain the calculated *count* and *sum–of–scores* evidence measures per function. The mean based evidence measures can be calculated from these two measures. The "Label" indicates wether the function is annotated in the reference database for *A. thaliana* by containing a value above zero. (ZIP 1 MB)

Additional file 4:**Evaluation of Scoring Methods.** ROC curves for the different scoring methods per sample and tool. All features were ranked according to the calculated evidence and performance is calculated for each rank. (PDF 356 KB)

Additional file 5:**Histograms of scores.** The distributions of the *mean–score* evidence measures are modeled by two Gaussian distributions and fitted by an unsupervised Expectation–Maximization algorithm. The *scaled mean–score* evidence values are modeled by two Gamma distributions. The evidence value histograms of the falsely predicted and the annotated functions are colored in red and green, respectively. The curves correspond to the probability distributions of the two component mixture model. Although the probability density curves are shown colored in the plot, the fitting of the model was performed in an unsupervised manner. Histograms were generated for all combinations of samples and tools. Even though the algorithms have converged to the maximum likelihood solution, the resulting models sometimes do not fit the observed data very well. (PDF 546 KB)

Additional file 6:**Performance for Gaussian model–based filtering on** ***mean–score*** **values.** Quality of the filtering for all tools regarding the *mean–scores*. True–Positive–Rate (TPR), False–Positive–Rate (FPR), Precision (Positive–Predictive–Values) and F1–Score (F1) were calculated after filtering the single samples. (PDF 28 KB)

Additional file 7:**Performance for Gamma model–based filtering on** ***scaled mean–score*** **values.** Quality of the filtering for all tools regarding the *scaled mean–scores*. True–Positive–Rate (TPR), False–Positive–Rate (FPR), Precision (Positive–Predictive–Values) and F1–Score (F1) were calculated after filtering the single samples. (PDF 28 KB)

Additional file 8:**Performance after filtering and different consensus thresholds.** To combine the samples, different consensus thresholds *c* were applied. A function was predicted present if contained in at least *c* samples after filtering the samples. See also Figures2 and3. (PDF 32 KB)

Additional file 9:**Prediction on** ***V. dahliae JR2.*** The scores and predictions calculated for *V. dahliae JR2* using the full KEGG database. (ZIP 399 KB)

Additional file 10:**Comparison of the predicted functions of** ***V. dahliae JR2.*** Venn diagram comparing the number of predicted functions from the transcriptome using the full database, after removing *V. alfalfae* and the genome, respectively. (PNG 26 KB)

## References

[1] Kanehisa M, Goto S (2000). KEGG: Kyoto Encyclopedia of Genes and Genomes. Nucleic Acids Res.

[2] Kanehisa M, Goto S, Sato Y, Furumichi M, Tanabe M (2012). KEGG for integration and interpretation of large-scale molecular data sets. Nucleic Acids Res.

[3] Caspi R, Foerster H, Fulcher CA, Kaipa P, Krummenacker M, Latendresse M, Paley S, Rhee SY, Shearer AG, Tissier C, Walk TC, Zhang P, Karp PD (2008). The MetaCyc database of metabolic pathways and enzymes and the BioCyc collection of Pathway/Genome databases. Nucleic Acids Res.

[4] Caspi R, Altman T, Dreher K, Fulcher CA, Subhraveti P, Keseler IM, Kothari A, Krummenacker M, Latendresse M, Mueller LA, Ong Q, Paley S, Pujar A, Shearer AG, Travers M, Weerasinghe D, Zhang P, Karp PD (2012). The MetaCyc database of metabolic pathways and enzymes and the BioCyc collection of Pathway/Genome Databases. Nucleic Acids Res.

[5] Karp PD, Paley S, Romero P (2002). The Pathway Tools software. Bioinformatics.

[6] Ye Y, Doak TG: **A parsimony approach to biological pathway reconstruction/inference for genomes and metagenomes.** *PLoS Comput Biol* 1000465.,**5**(8)**:** doi:10.1371/journal.pcbi.1000465 10.1371/journal.pcbi.1000465 PMC2714467 19680427

[7] Henry CS, DeJongh M, Best AA, Frybarger PM, Linsay B, Stevens RL (2010). High-throughput generation, optimization and analysis of genome-scale metabolic models. Nat Biotechnol.

[8] Metzker ML (2010). Sequencing technologies - the next generation. Nat Rev Genet.

[9] Zerbino DR, Birney E (2008). Velvet: algorithms for de novo short read assembly using de bruijn graphs. Genome Res.

[10] Simpson JT, Wong K, Jackman SD, Schein JE, Jones SJM, Birol I (2009). ABySS: a parallel assembler for short read sequence data. Genome Res.

[11] Peng Y, Leung HCM, Yiu SM, Chin FYL (2012). IDBA-UD: a de novo assembler for single-cell and metagenomic sequencing data with highly uneven depth. Bioinformatics.

[12] Altschul SF, Madden TL, Schäffer AA, Zhang J, Zhang Z, Miller W, Lipman DJ (1997). Gapped BLAST and PSI-BLAST: a new generation of protein database search programs. Nucleic Acids Res.

[13] Earl D, Bradnam K, John JS, Darling A, Lin D, Fass J, Yu HOK, Buffalo V, Zerbino DR, Diekhans M, Nguyen N, Ariyaratne PN, Sung W-K, Ning Z, Haimel M, Simpson JT, Fonseca NA, Birol I, Docking TR, Ho IY, Rokhsar DS, Chikhi R, Lavenier D, Chapuis G, Naquin D, Maillet N, Schatz MC, Kelley DR, Phillippy AM, Koren S (2011). Assemblathon 1: a competitive assessment of de novo short read assembly methods. Genome Res.

[14] Bradnam KR, Fass JN, Alexandrov A, Baranay P, Bechner M, Birol I, Boisvert S, Chapman JA, Chapuis G, Chikhi R, Chitsaz H, Chou W-C, Corbeil J, Fabbro CD, Docking TR, Durbin R, Earl D, Emrich S, Fedotov P, Fonseca NA, Ganapathy G, Gibbs RA, Gnerre S, Godzaridis E, Goldstein S, Haimel M, Hall G, Haussler D, Hiatt JB, Ho IY (2013). Assemblathon 2: evaluating de novo methods of genome assembly in three vertebrate species. Gigascience.

[15] Pellicer J, Fay MF, Leitch IJ (2010). The largest eukaryotic genome of them all?. Bot J Linnean Soc.

[16] Gross B, Rieseberg L (2005). The ecological genetics of homoploid hybrid speciation. J Hered.

[17] Mallet J (2007). Hybrid speciation. Nature.

[18] English AC, Richards S, Han Y, Wang M, Vee V, Qu J, Qin X, Muzny DM, Reid JG, Worley KC, Gibbs RA (2012). Mind the gap: upgrading genomes with Pacific Biosciences RS long-read sequencing technology.gerstein. PLoS One.

[19] Wang Z, Gerstein M, Snyder M (2009). RNA-Seq: a revolutionary tool for transcriptomics. Nat Rev Genet.

[20] Koonin EV, Fedorova ND, Jackson JD, Jacobs AR, Krylov DM, Makarova KS, Mazumder R, Mekhedov SL, Nikolskaya AN, Rao BS, Rogozin IB, Smirnov S, Sorokin AV, Sverdlov AV, Vasudevan S, Wolf YI, Yin JJ, Natale DA (2004). A comprehensive evolutionary classification of proteins encoded in complete eukaryotic genomes. Genome Biol.

[21] Birol I, Jackman SD, Nielsen CB, Qian JQ, Varhol R, Stazyk G, Morin RD, Zhao Y, Hirst M, Schein JE, Horsman DE, Connors JM, Gascoyne RD, Marra MA, Jones SJM (2009). De novo transcriptome assembly with abyss. Bioinformatics.

[22] Peng Y, Leung HCM, Yiu S-M, Lv M-J, Zhu X-G, Chin FYL (2013). IDBA-tran: a more robust de novo de bruijn graph assembler for transcriptomes with uneven expression levelspis. Bioinformatics.

[23] Ye Y, Choi J-H, Tang H (2011). RAPSearch: a fast protein similarity search tool for short reads. BMC Bioinformatics.

[24] Huson DH, Xie C (2013). A poor man’s BLASTX - high-throughput metagenomic protein database search using PAUDA. Bioinformatics.

[25] Murphy LR, Wallqvist A, Levy RM (2000). Simplified amino acid alphabets for protein fold recognition and implications for folding. Protein Eng.

[26] Meinicke P: **UProC: tools for ultra-fast protein domain classification.** [http://uproc.gobics.de]], Accessed April 2013 10.1093/bioinformatics/btu843 PMC4410661 25540185

[27] Dempster AP, Laird NM, Rubin DB (1977). Maximum likelihood from incomplete data via the EM algorithm. J R Stat Soc.

[28] Celeux G, Govaert G (1992). A classification EM algorithm for clustering and two stochastic versions. Comput Stat Data Anal.

[29] Celeux G, Diebolt J (1985). The SEM algorithm: a probabilistic teacher algorithm derived from the EM algorithm for the mixture problem. Comput Stat Q.

[30] Marquez Y, Brown JWS, Simpson C, Barta A, Kalyna M (2012). Transcriptome survey reveals increased complexity of the alternative splicing landscape in Arabidopsis. Genome Res.

[31] NCBI Sequence Read Archive: **Illumina RNA-Seq of Arabidopsis Col-0 to determine Alternative splicing landscape.** [https://www.ncbi.nlm.nih.gov/sra/SRX103665], Accessed April 2013

[32] Schmieder R, Edwards R (2011). Quality control and preprocessing of metagenomic datasets. Bioinformatics.

[33] Langmead B, Salzberg SL (2012). Fast gapped-read alignment with Bowtie 2. Nat Med.

[34] Fradin EF, Zhang Z, Rovenich H, Song Y, Liebrand TWH, Masini L, van den Berg GCM, Joosten MHAJ, Thomma BPHJ (2014). Functional analysis of the tomato immune receptor Ve1 through domain swaps with its non-functional homolog Ve2. PLoS ONE.

[35] Tran V-T, Braus-Stromeyer SA, Kusch H, Reusche M, Kaever A, Kühn A, Valerius O, Landesfeind M, Aßhauer K, Tech M, Hoff K, Pena-Centeno T, Stanke M, Lipka V, Braus GH (2014). Verticillium transcription activator of adhesion vta2 suppresses microsclerotia formation and is required for systemic infection of plant roots. New Phytol.

[36] Chatterji S, Yamazaki I, Bai Z, Eisen JA (2008). CompostBin: a DNA, composition-based algorithm for binning environmental shotgun reads. Research in Computational Molecular Biology.

[37] Kislyuk A, Bhatnagar S, Dushoff J, Weitz JS (2009). Unsupervised statistical clustering of environmental shotgun sequences. BMC Bioinformatics.

[38] Tanaseichuk O, Borneman J, Jiang T (2012). Separating metagenomic short reads into genomes via clustering. Algorithms Mol Biol.

[39] Jenkinson AF (1955). The frequency distribution of the annual maximum (or minimum) values of meteorological elements. Q J R Meteorol Soc.

