# Supplementary figures and images for: Predicting the functional repertoire of an organism from unassembled RNA–seq data
Source: BMC Genomics. 2014 Nov 20;15(1):1003. doi: 10.1186/1471-2164-15-1003 (PMC4258056; doi:10.1186/1471-2164-15-1003)

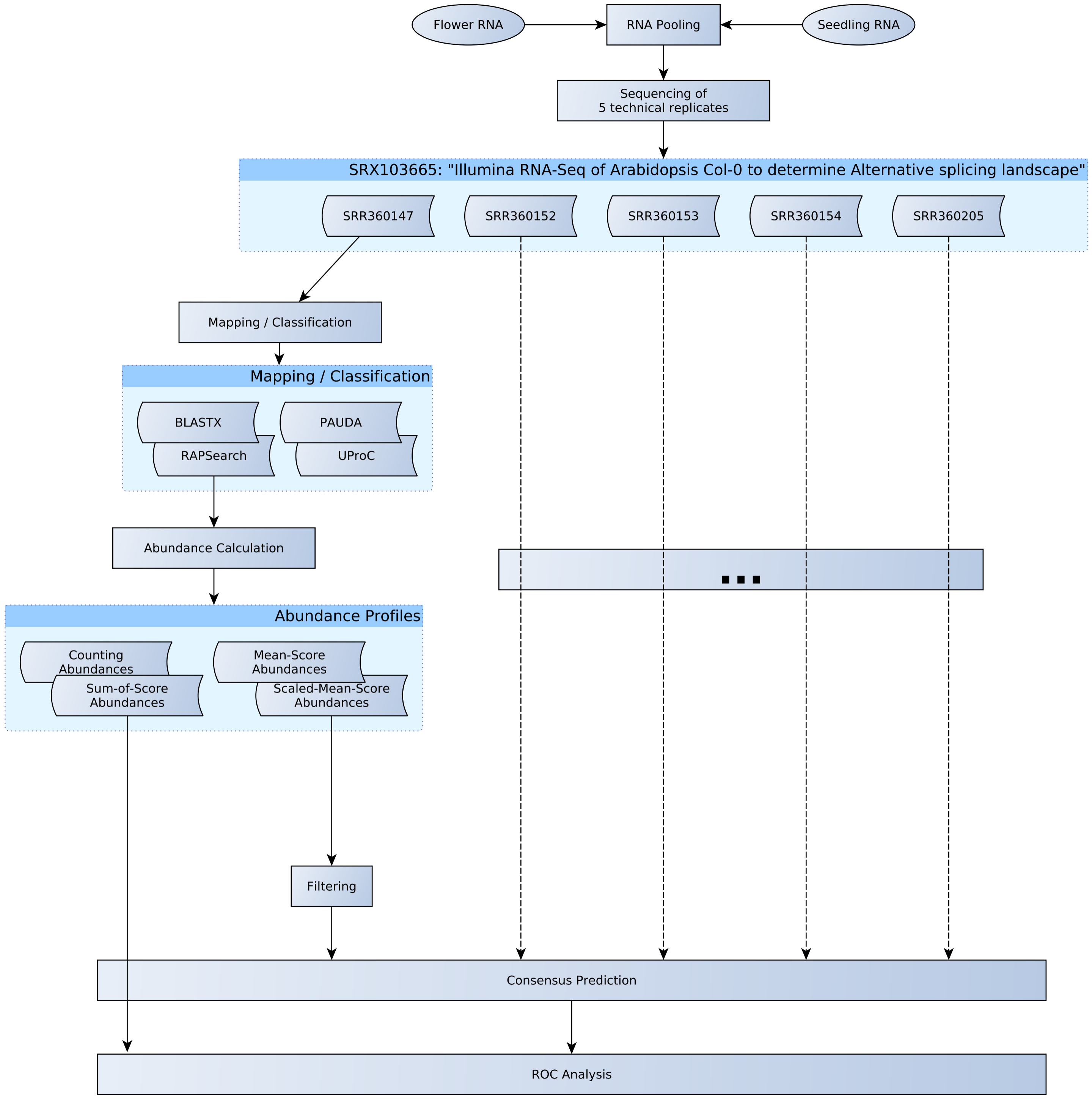

Supplement: Supplementary file 1 — Additional file 1:Work flow of the study. Visualization of the work-flow for this study. (PDF 130 KB) [file 12864_2014_6719_MOESM1_ESM.pdf]

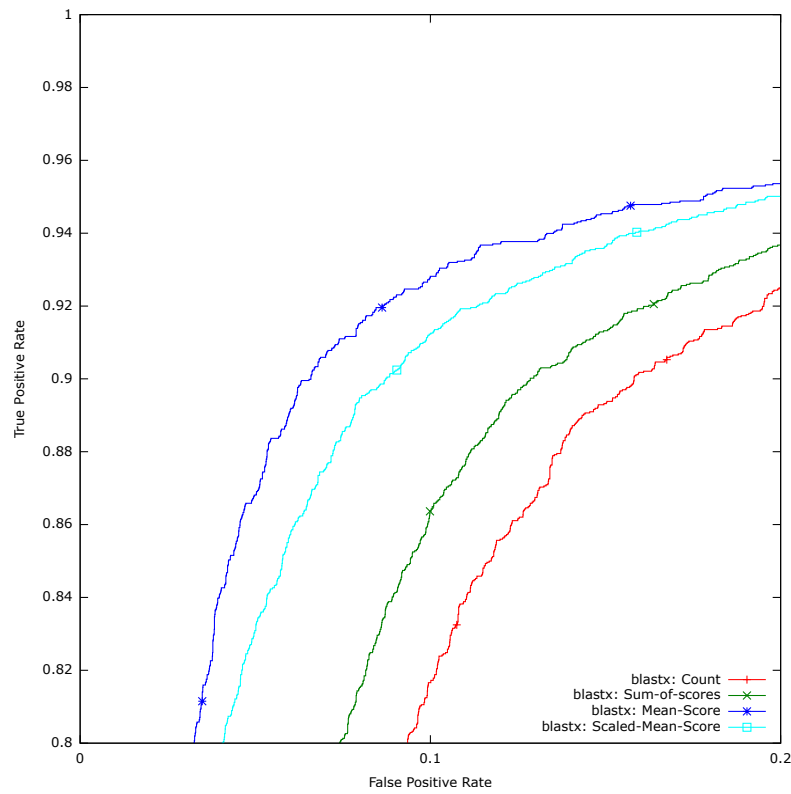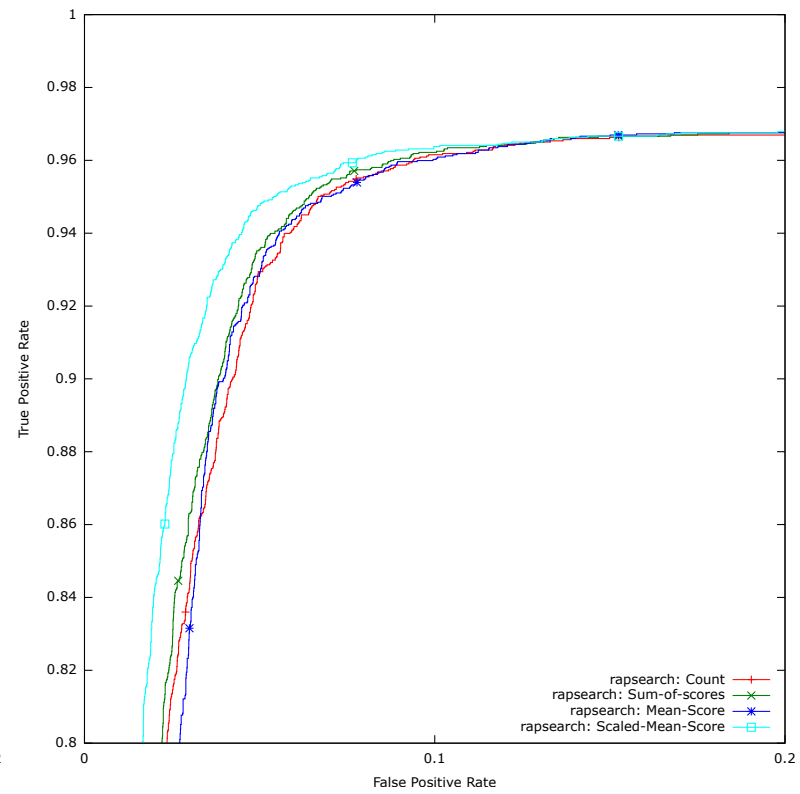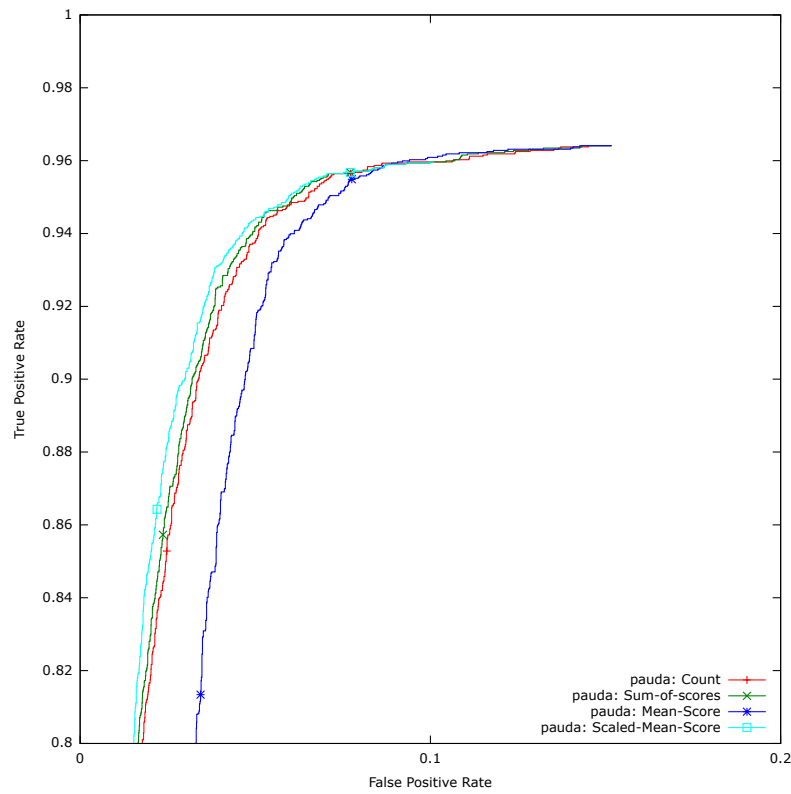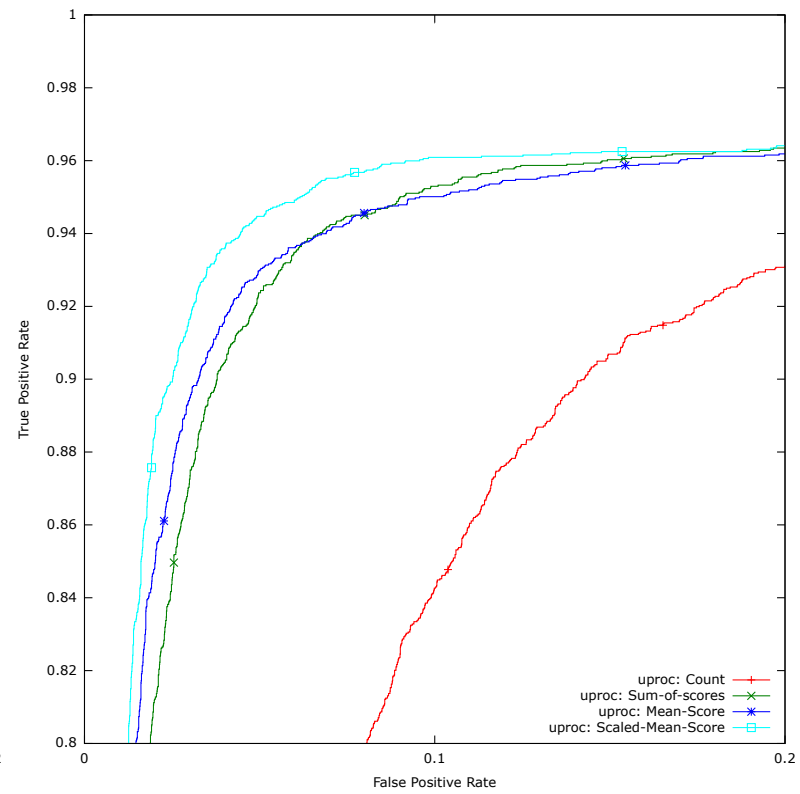

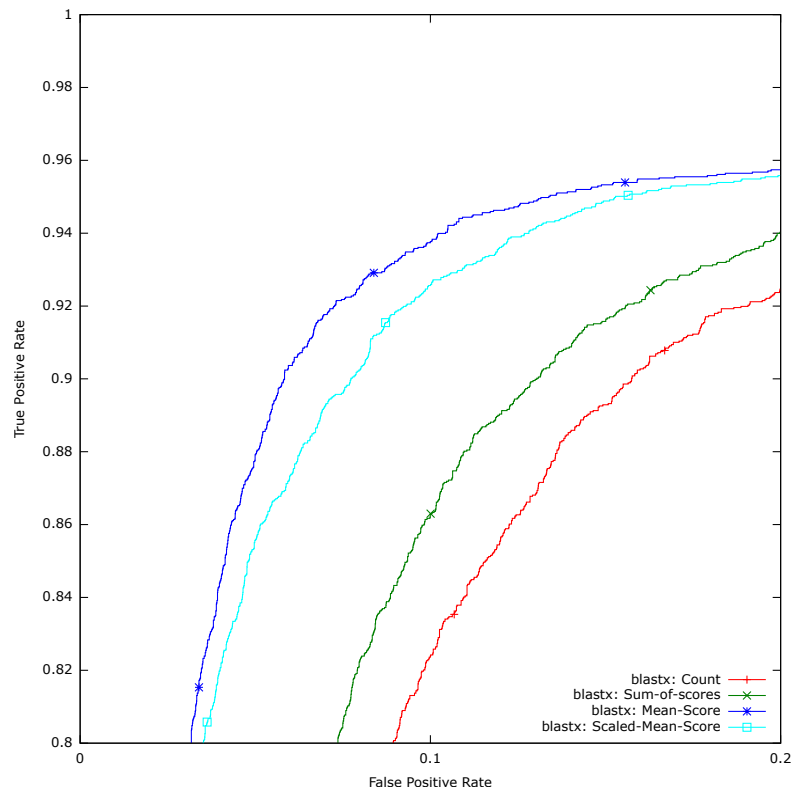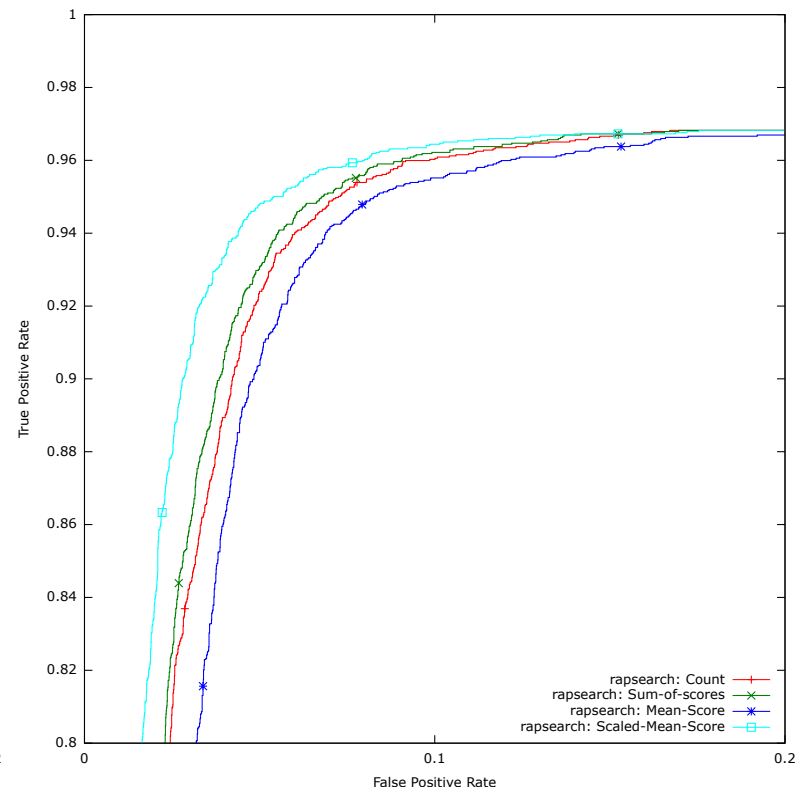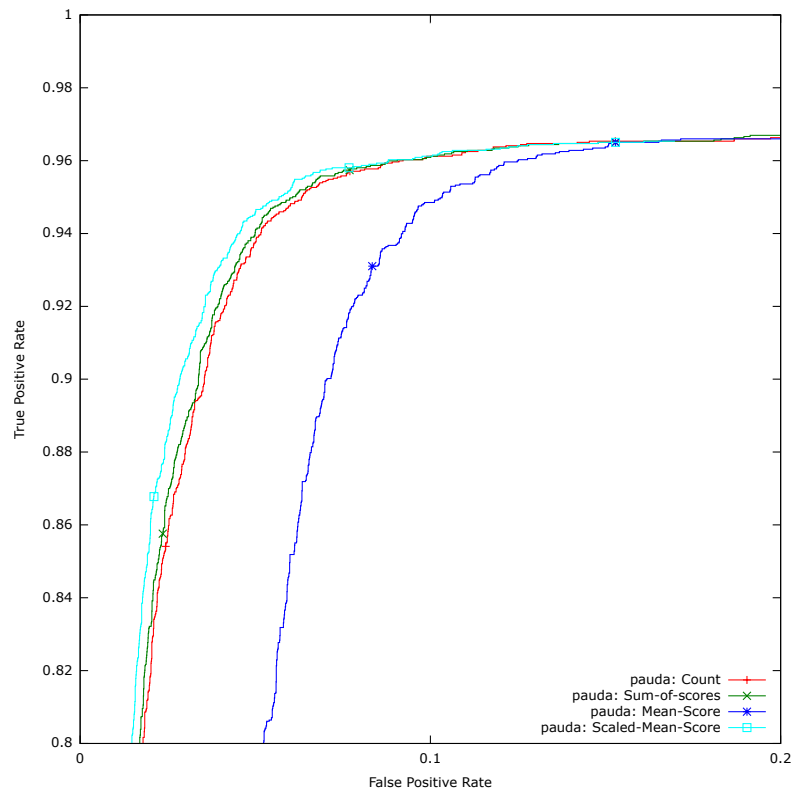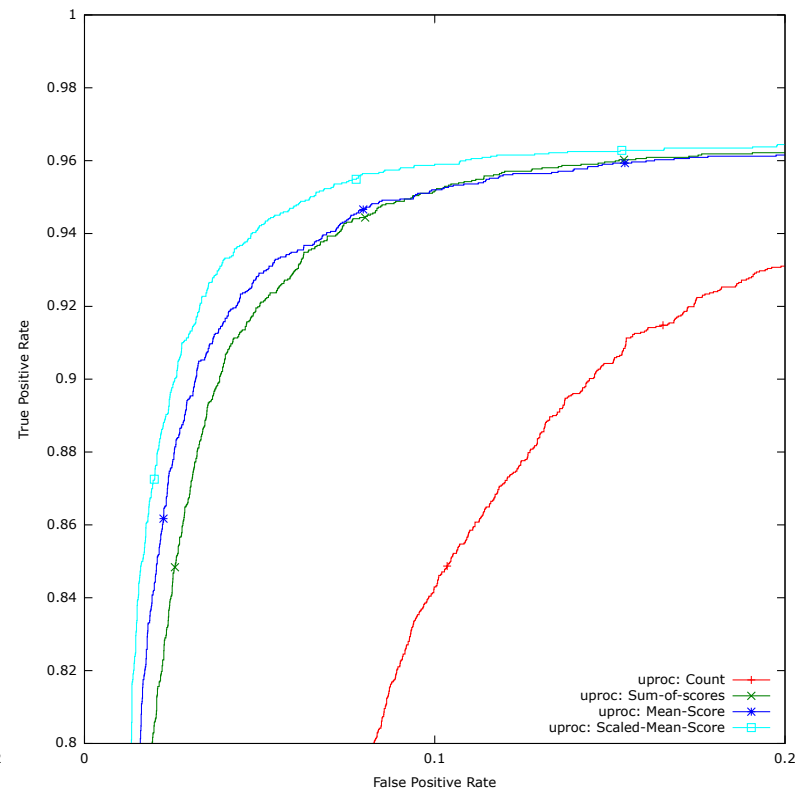

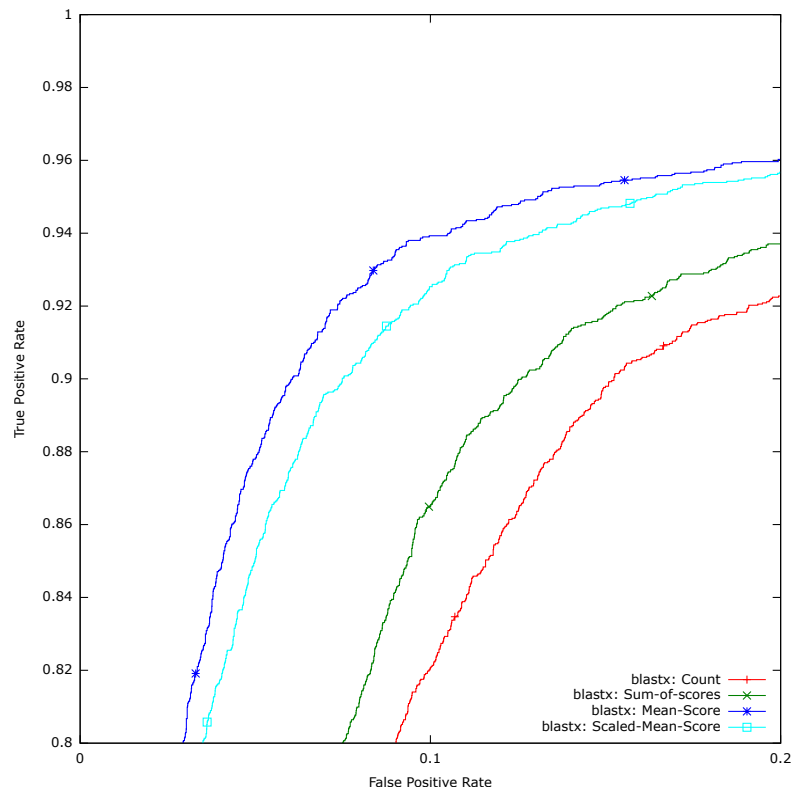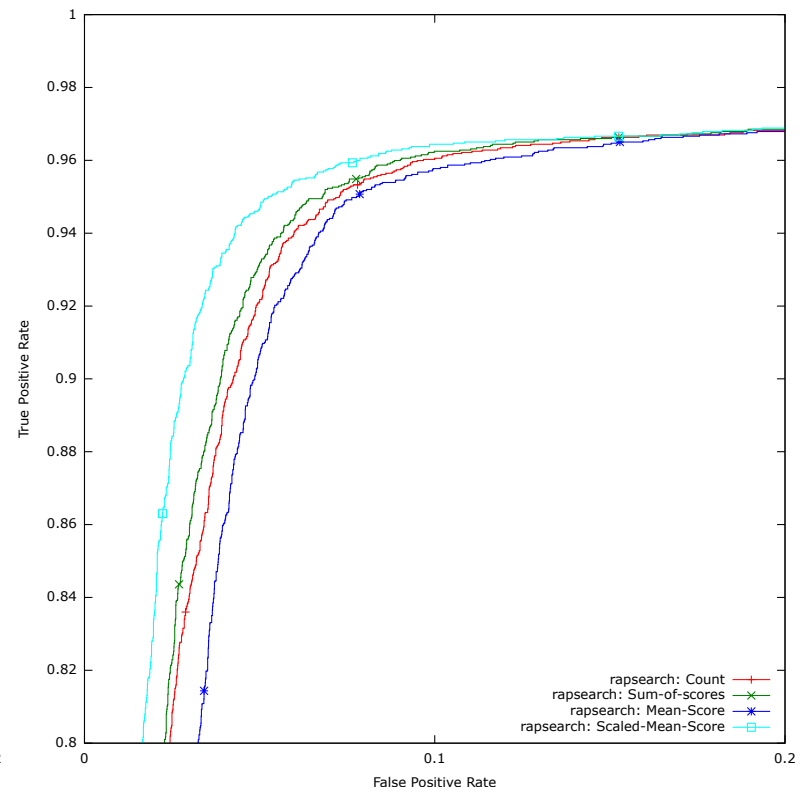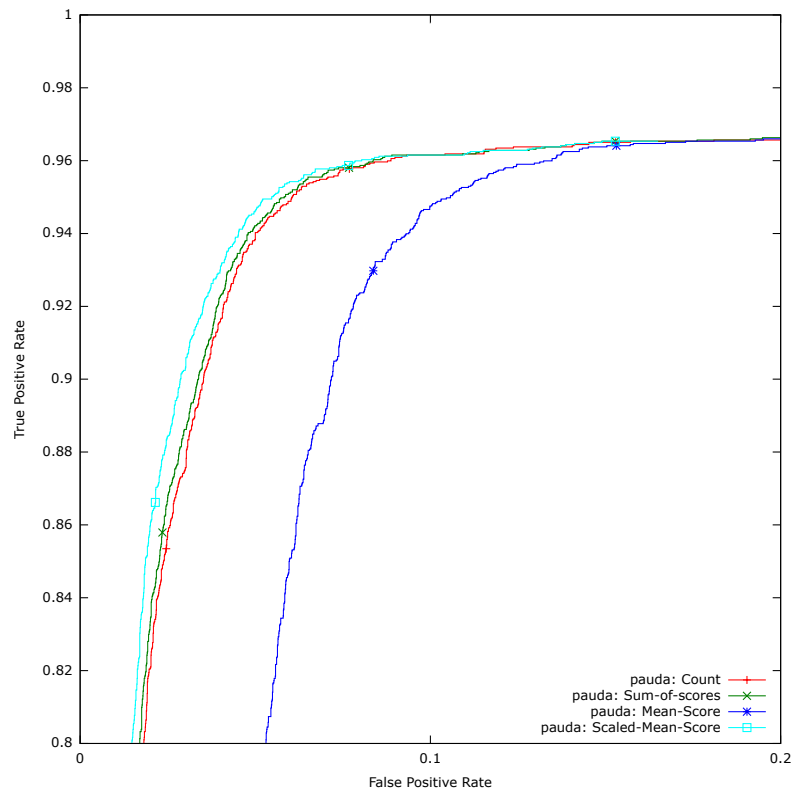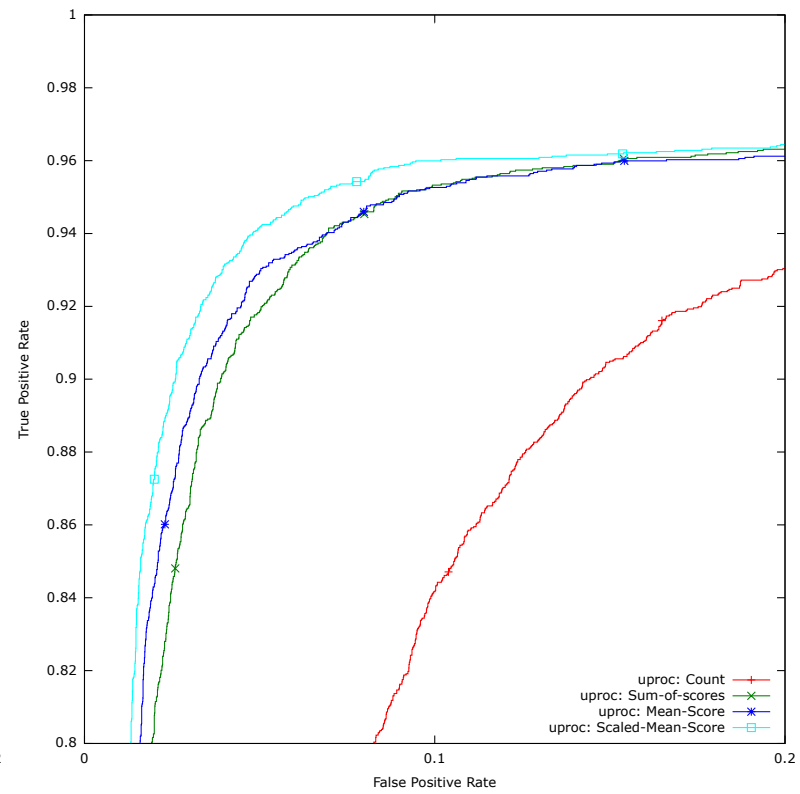

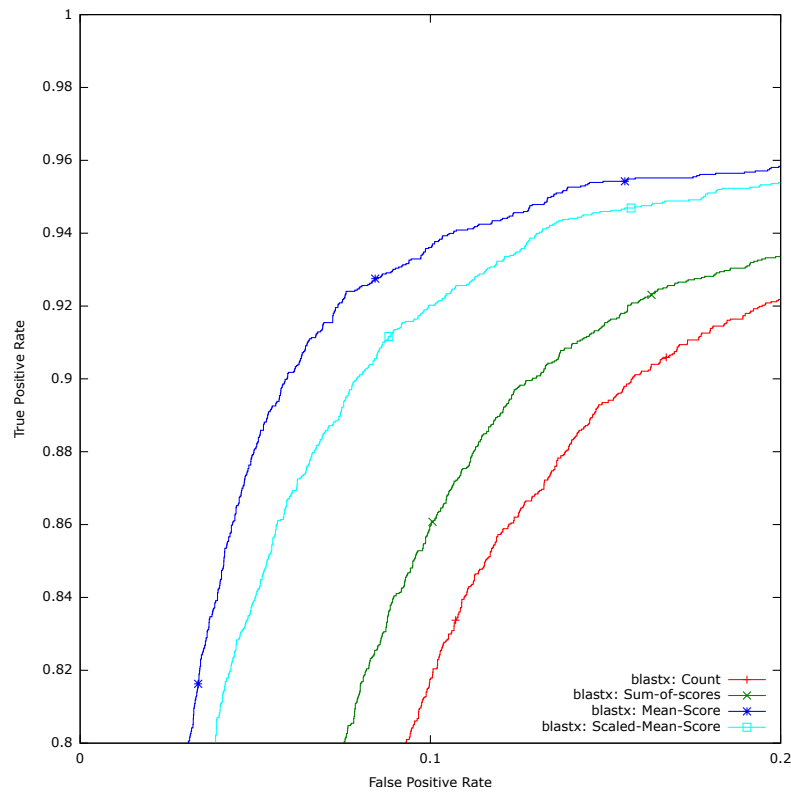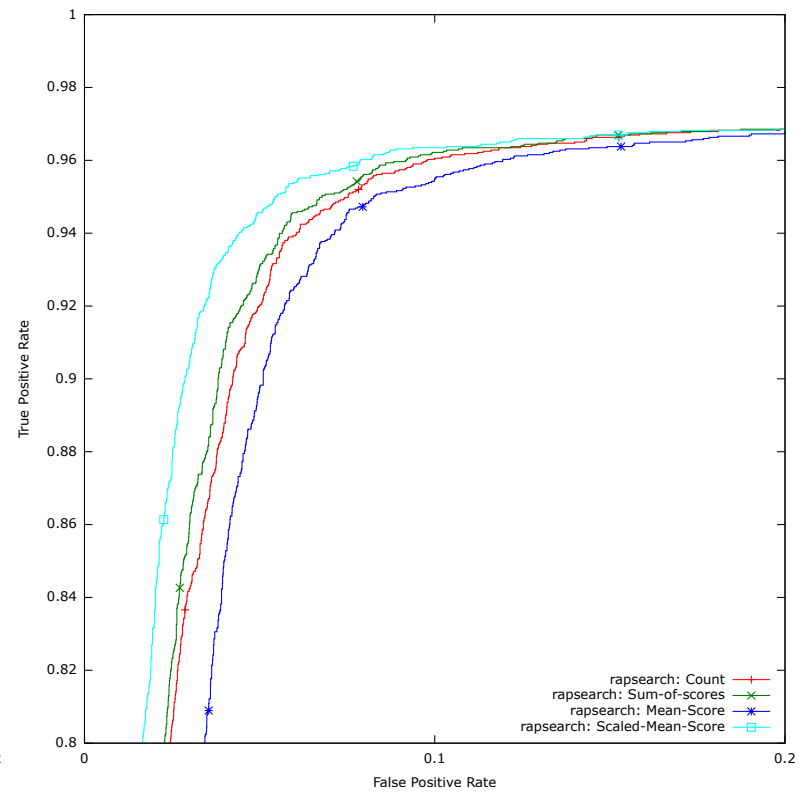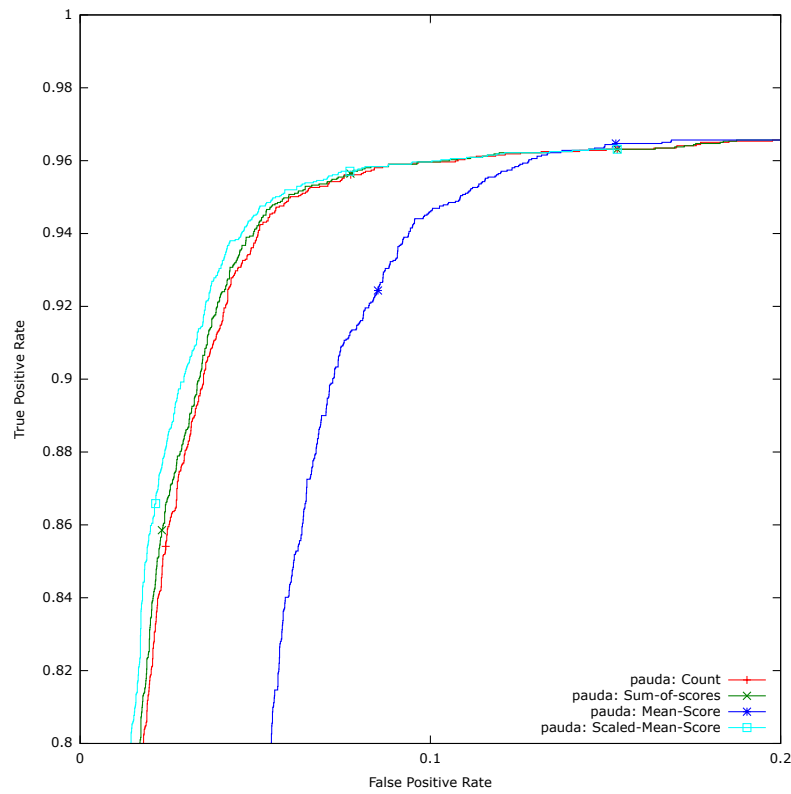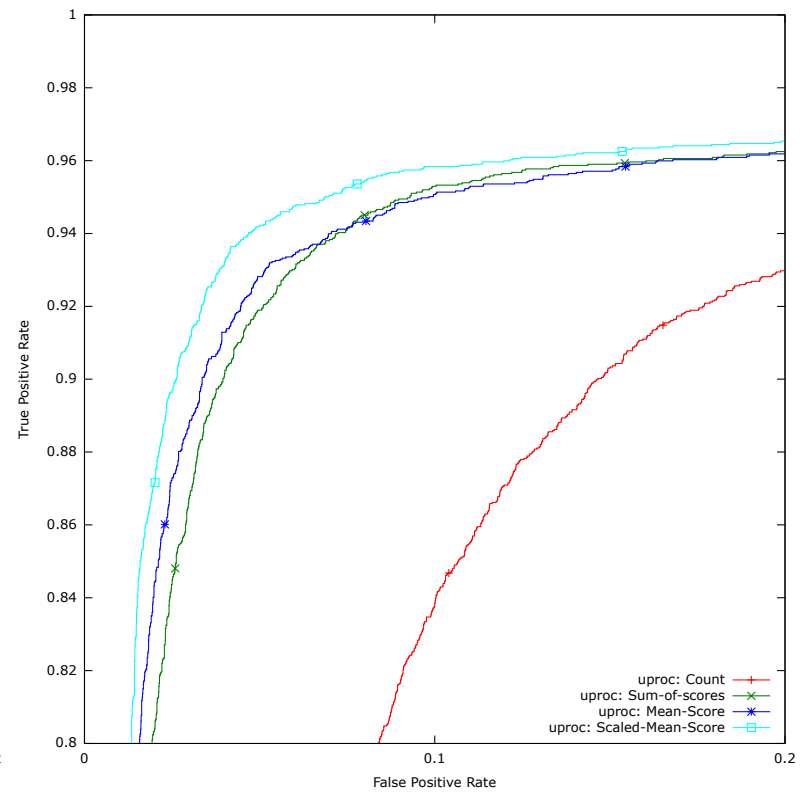

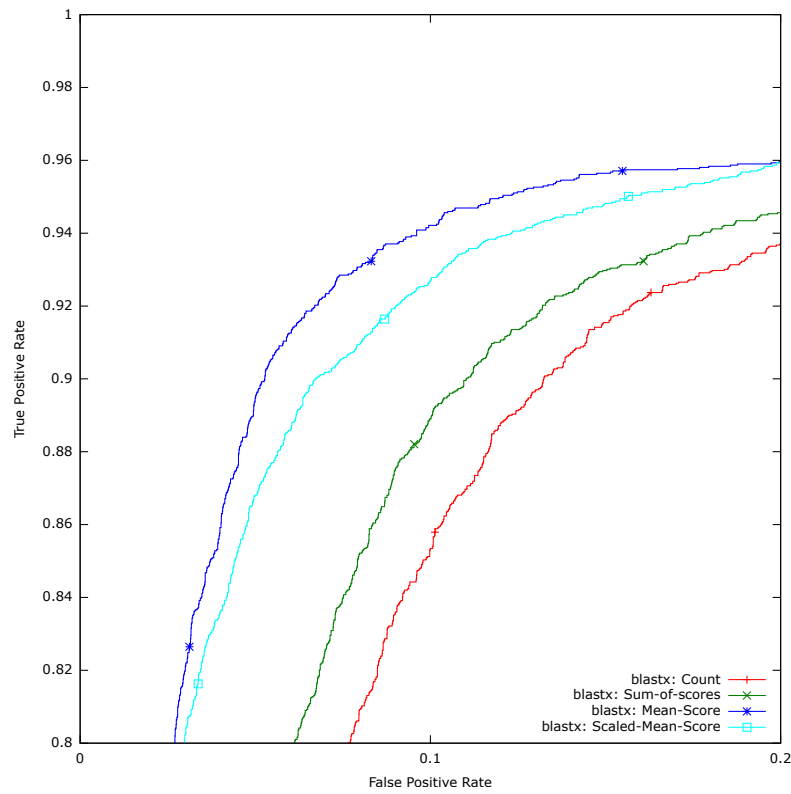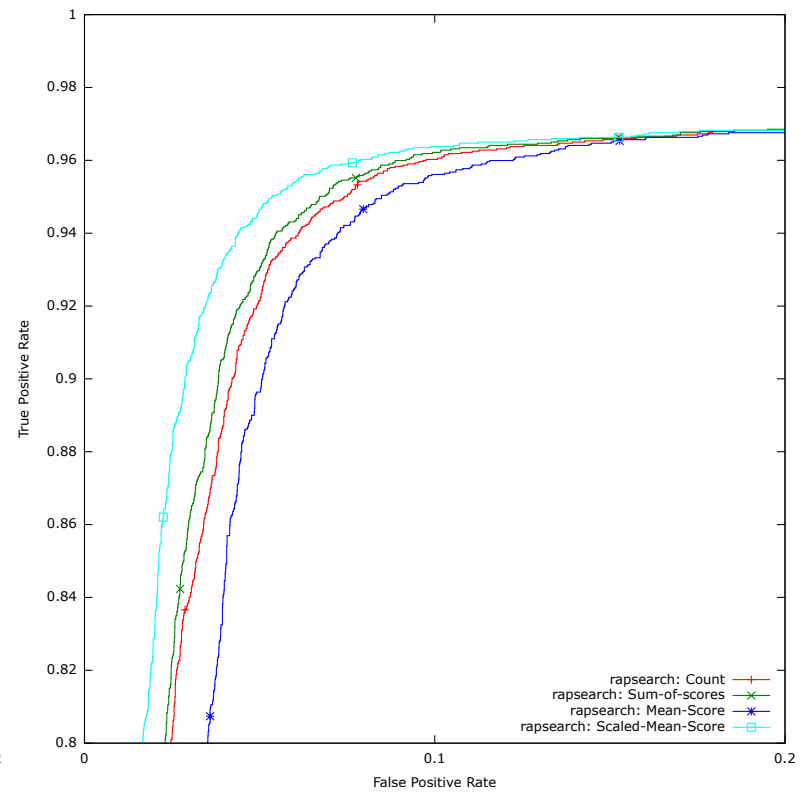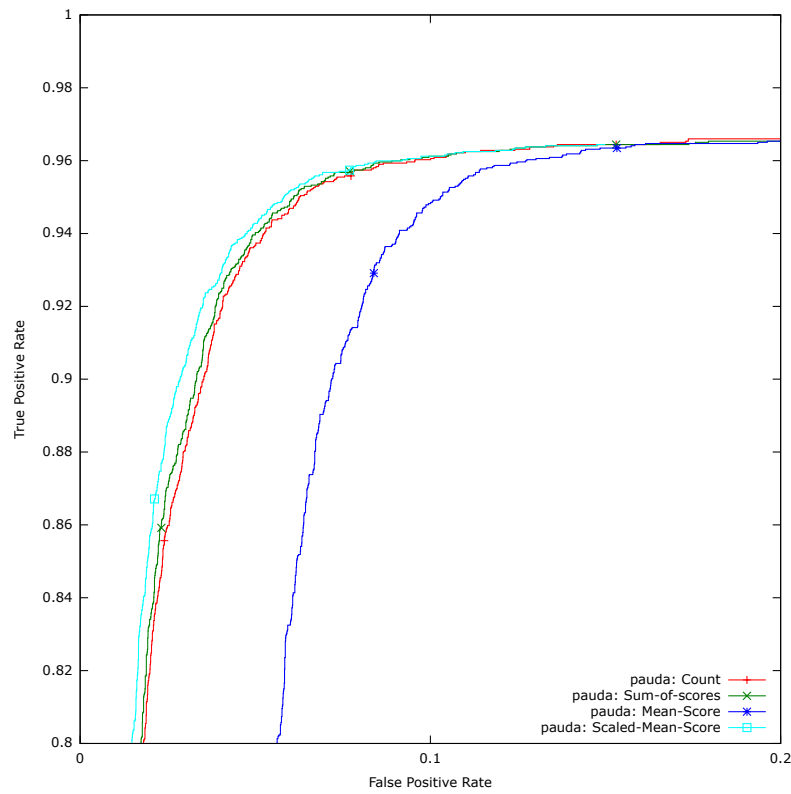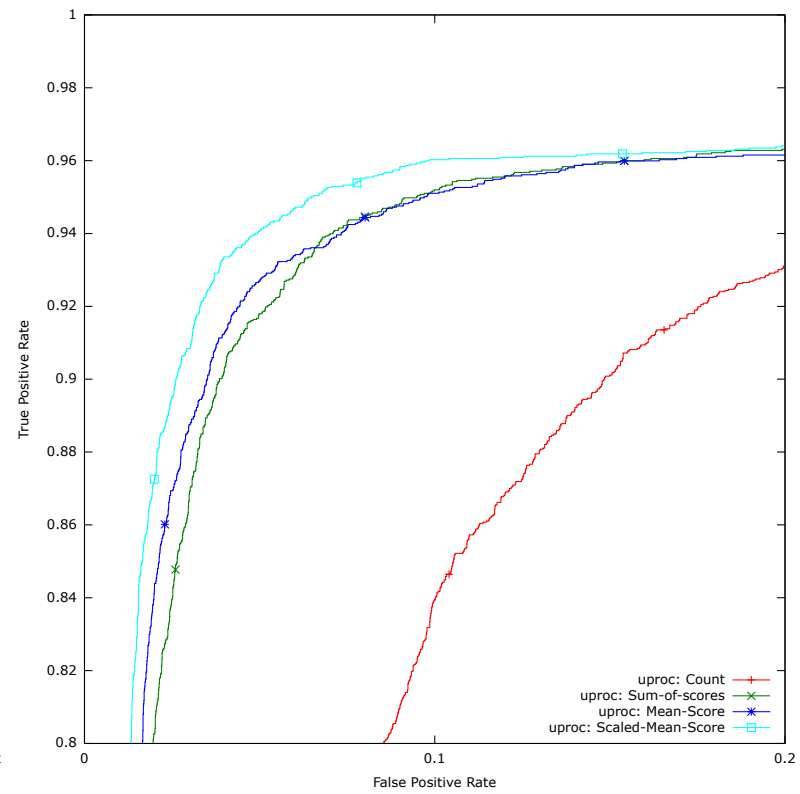

Supplement: Supplementary file 4 — Additional file 4:Evaluation of Scoring Methods. ROC curves for the different scoring methods per sample and tool. All features were ranked according to the calculated evidence and performance is calculated for each rank. (PDF 356 KB) [file 12864_2014_6719_MOESM4_ESM.pdf]

# Histograms for Mean–Score evidence values

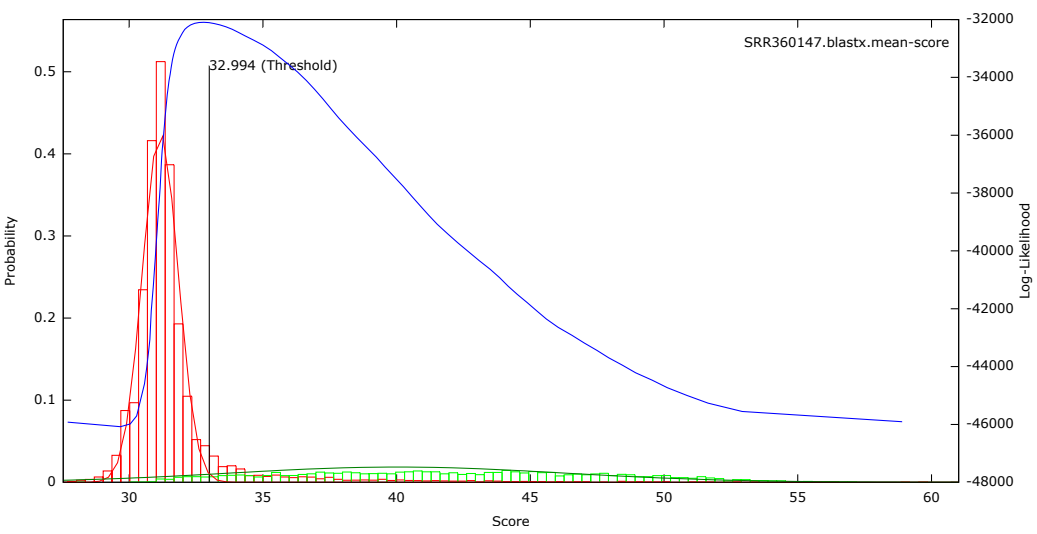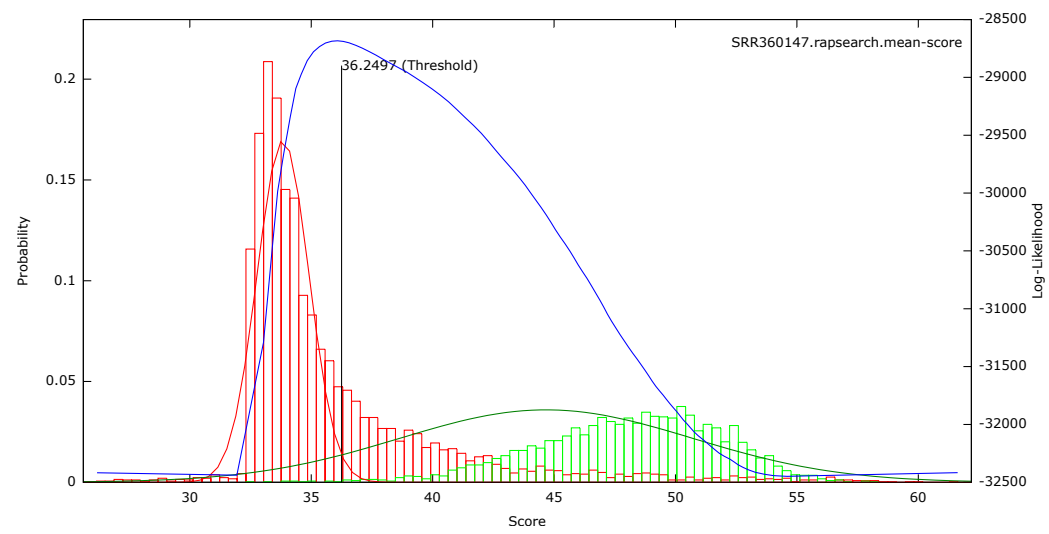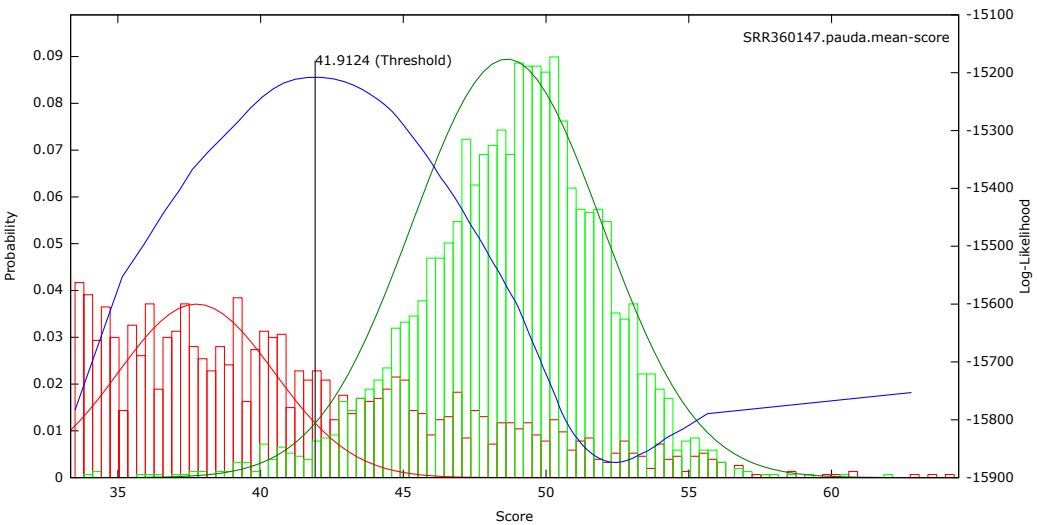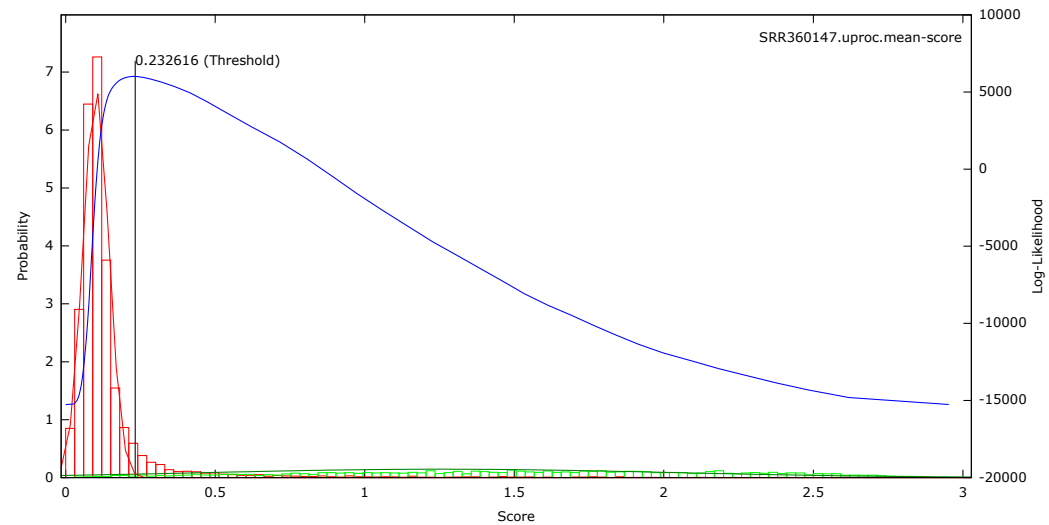

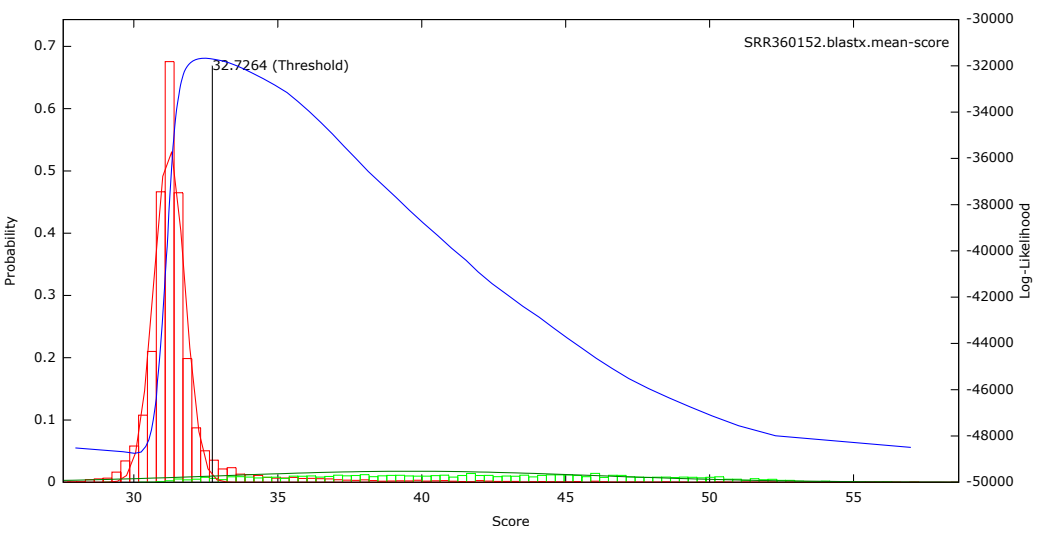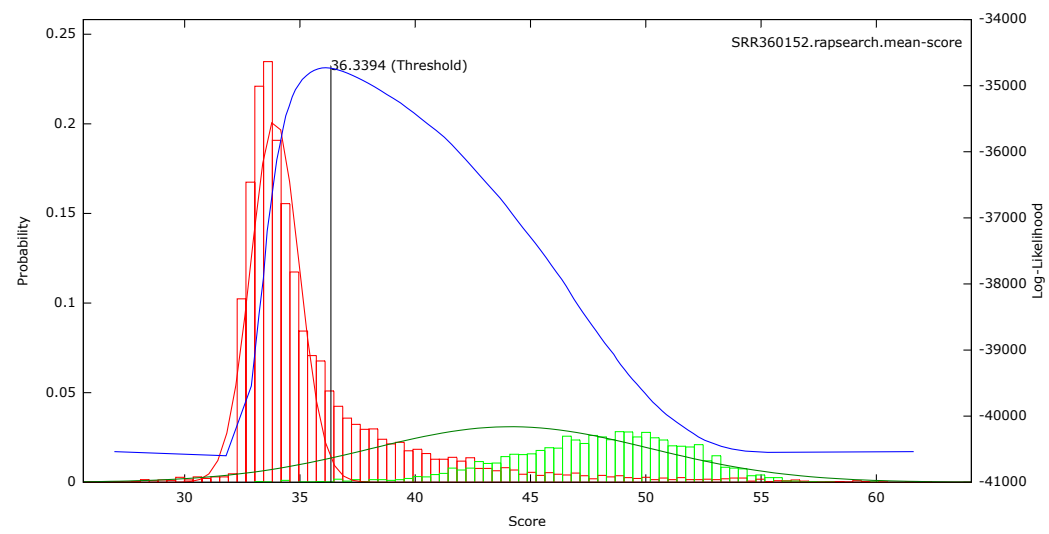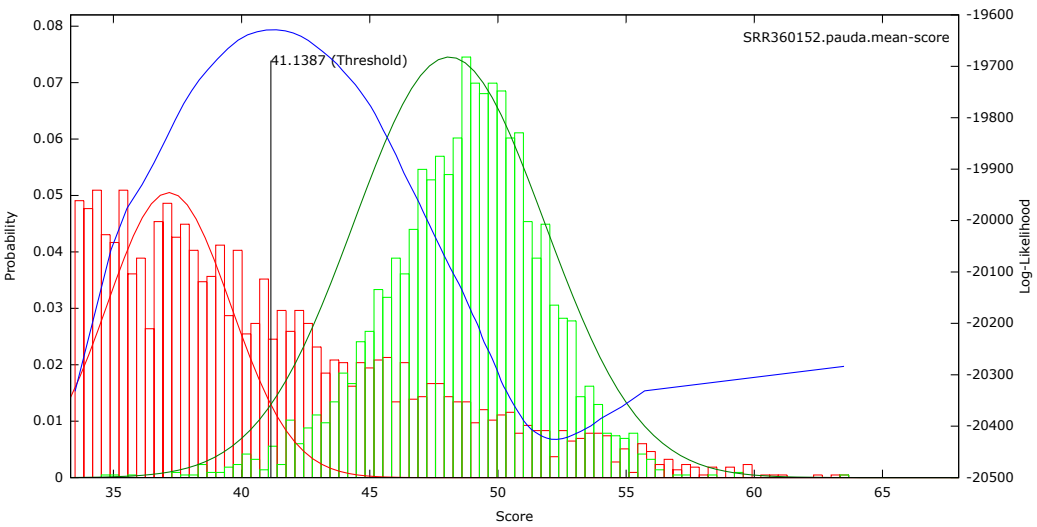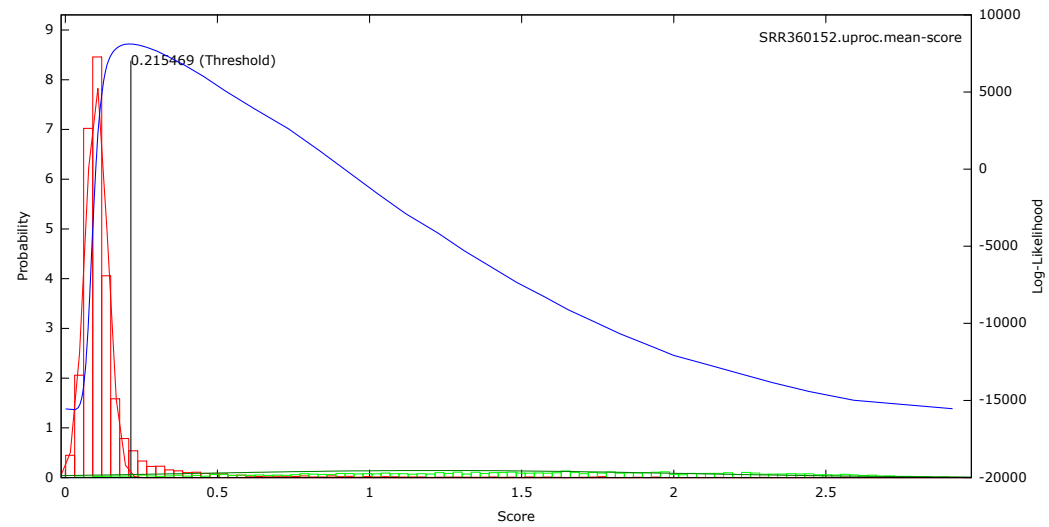

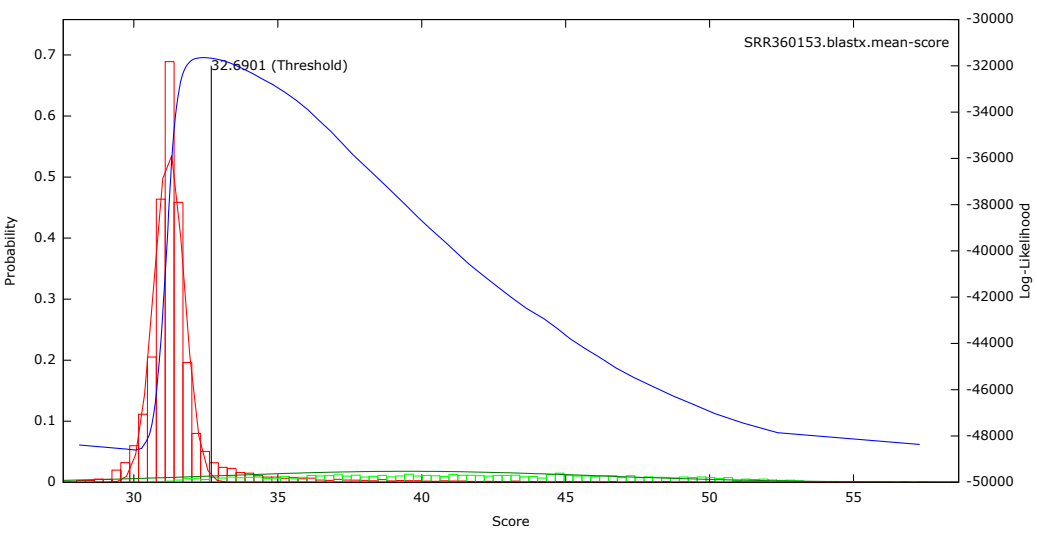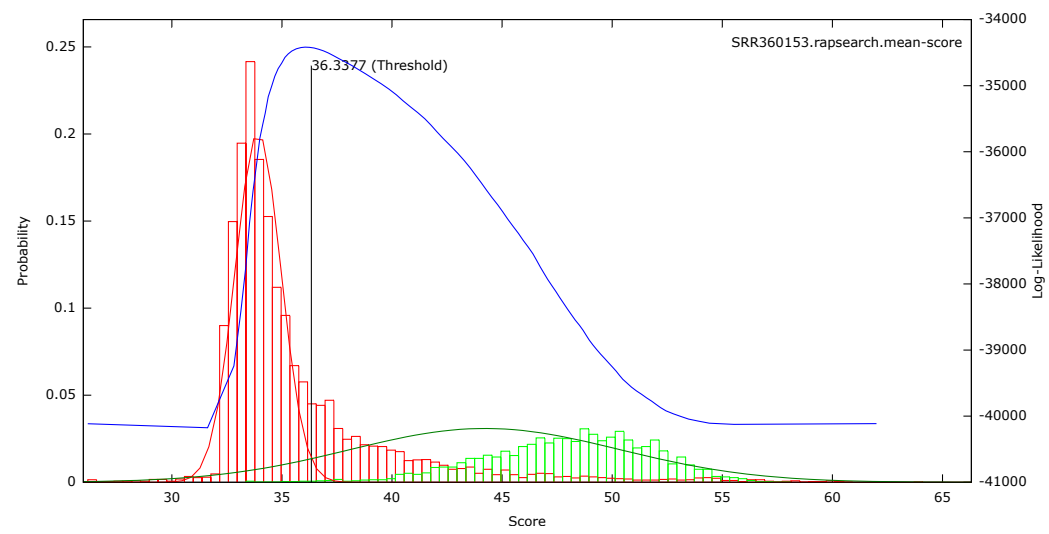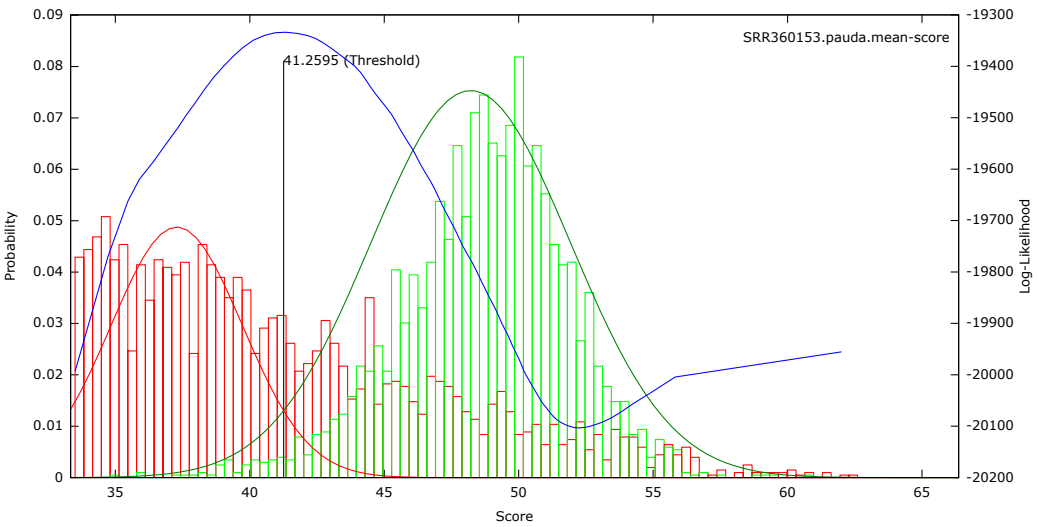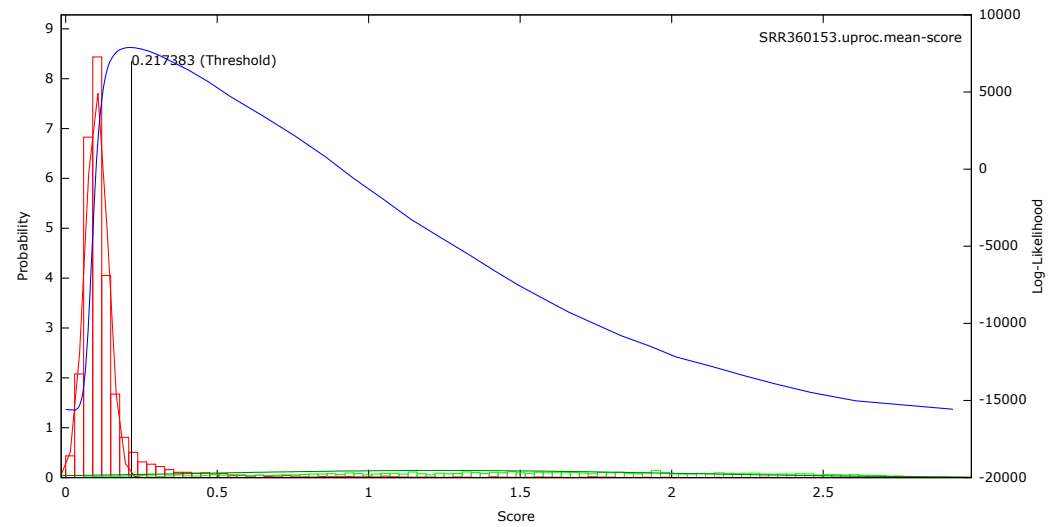

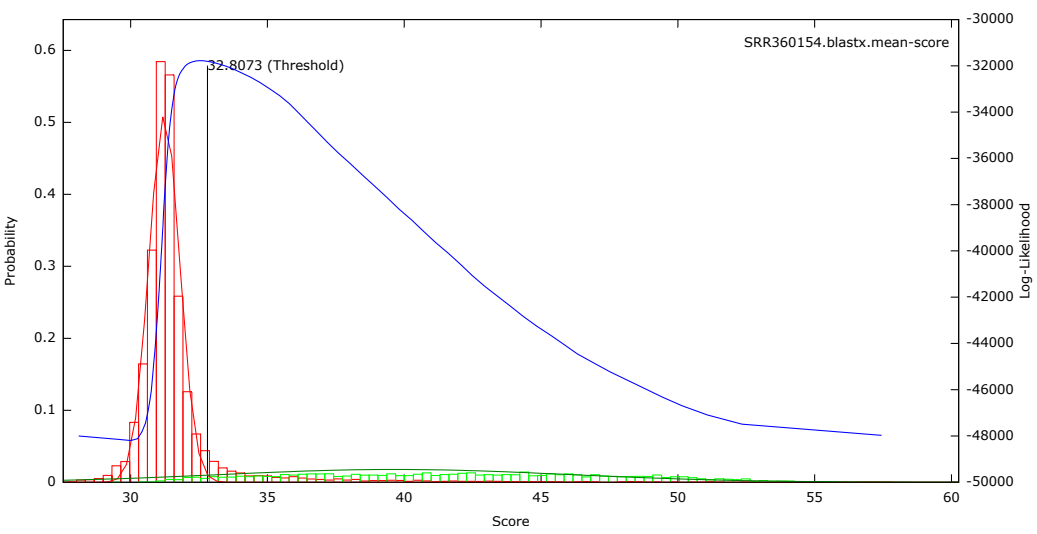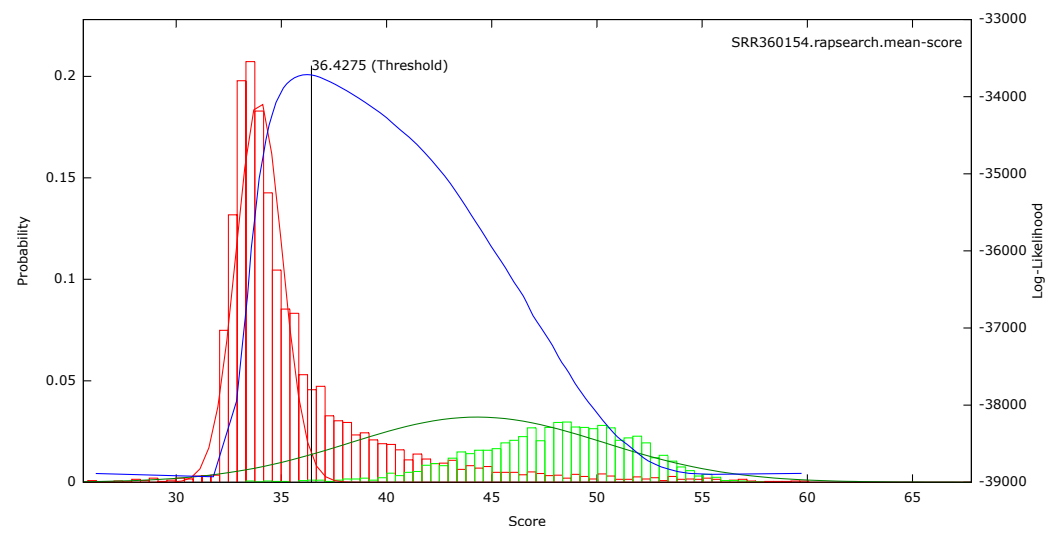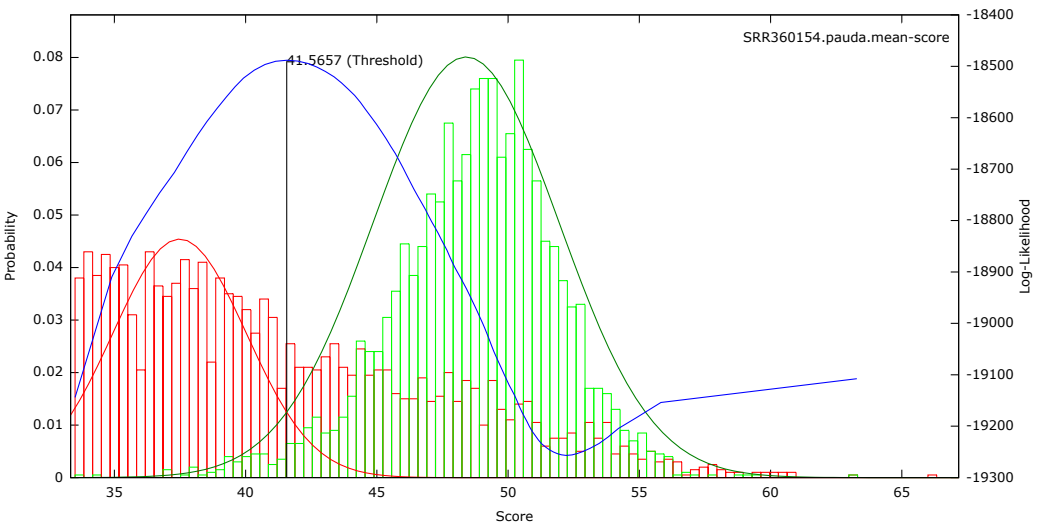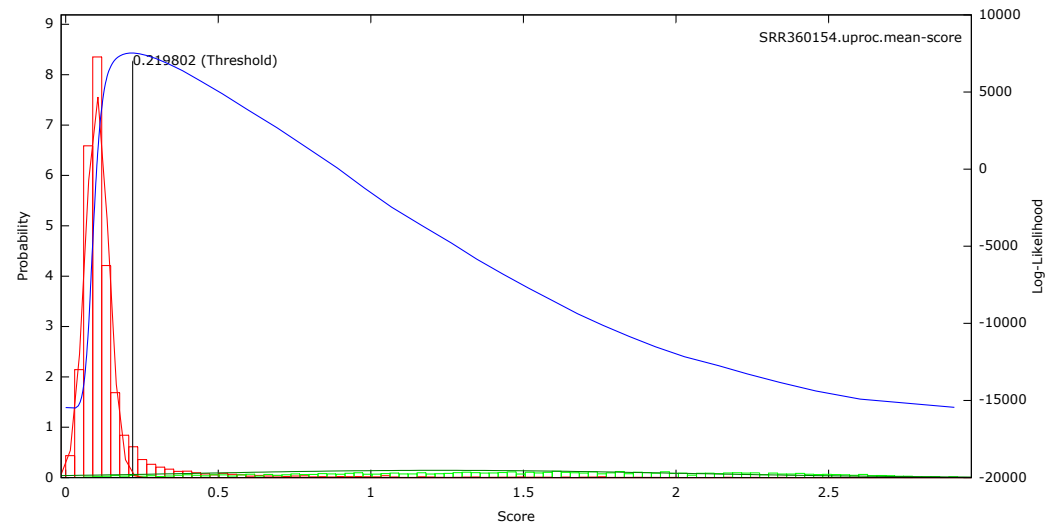

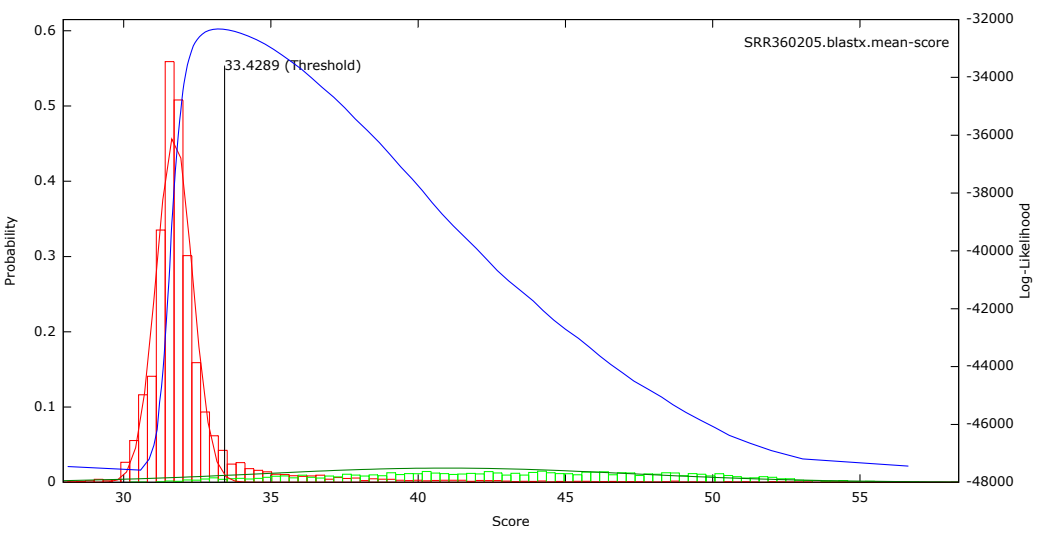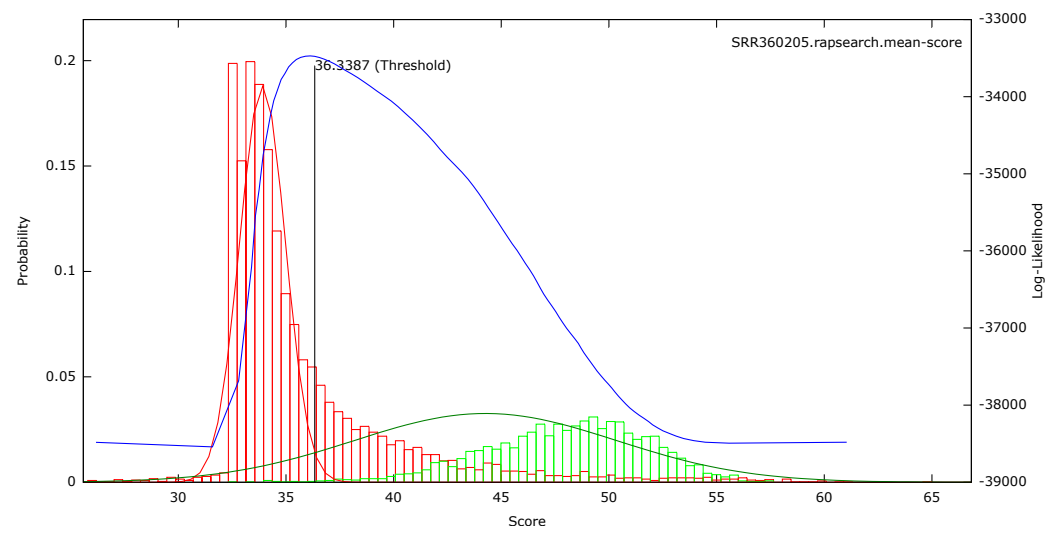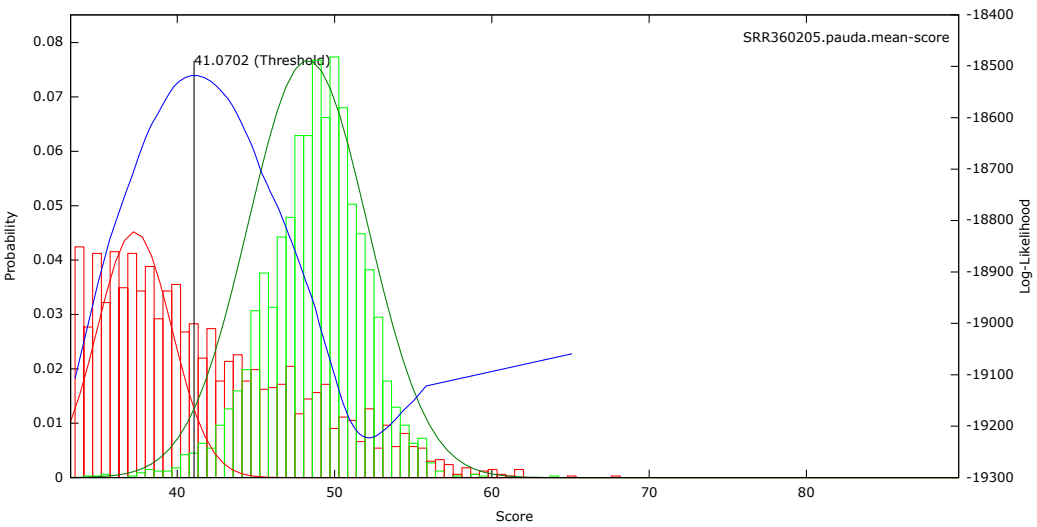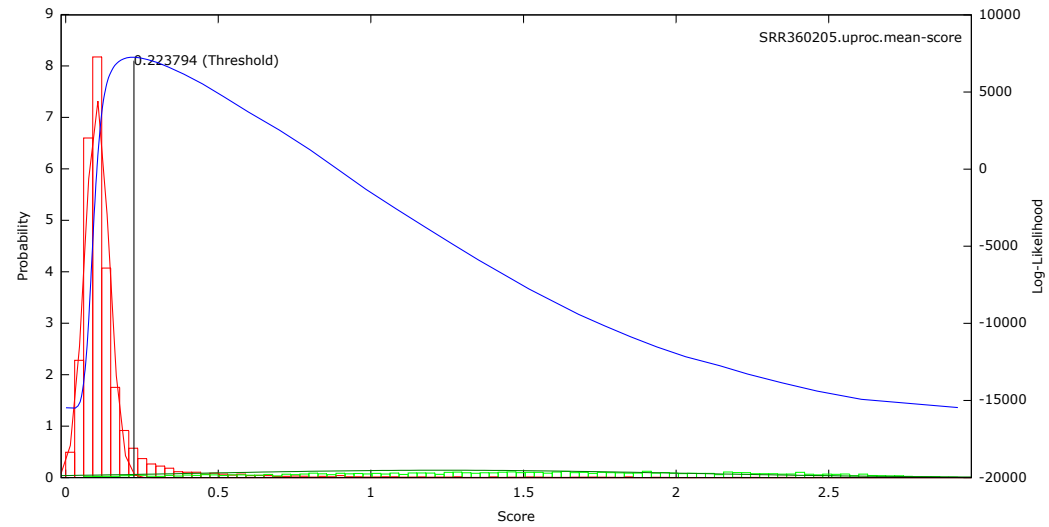

# Histograms for Scaled-Mean-Score evidence values

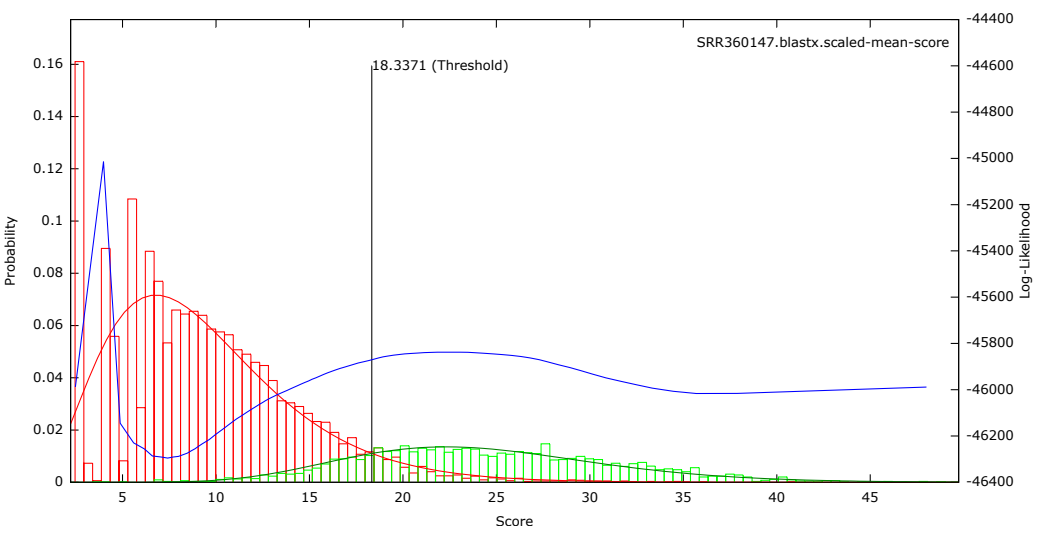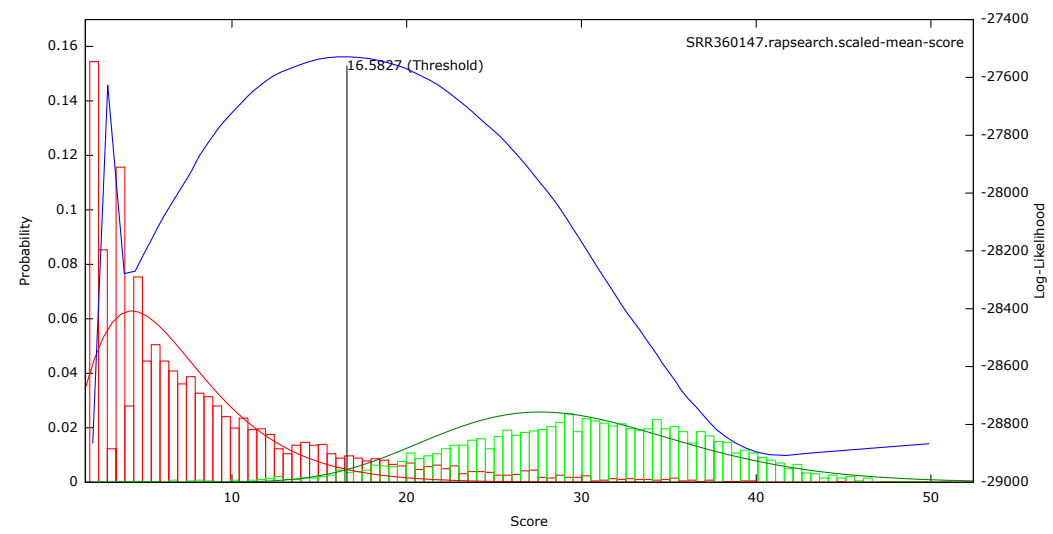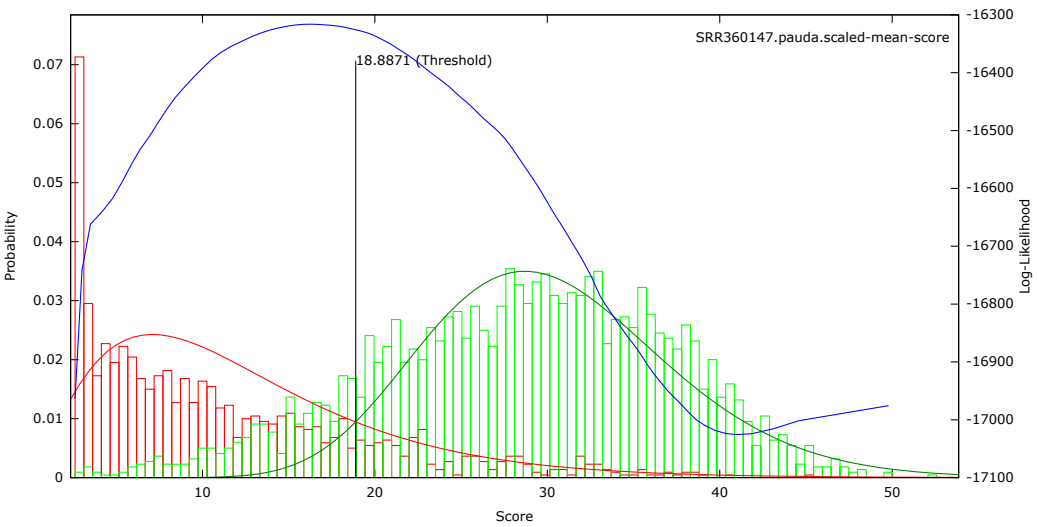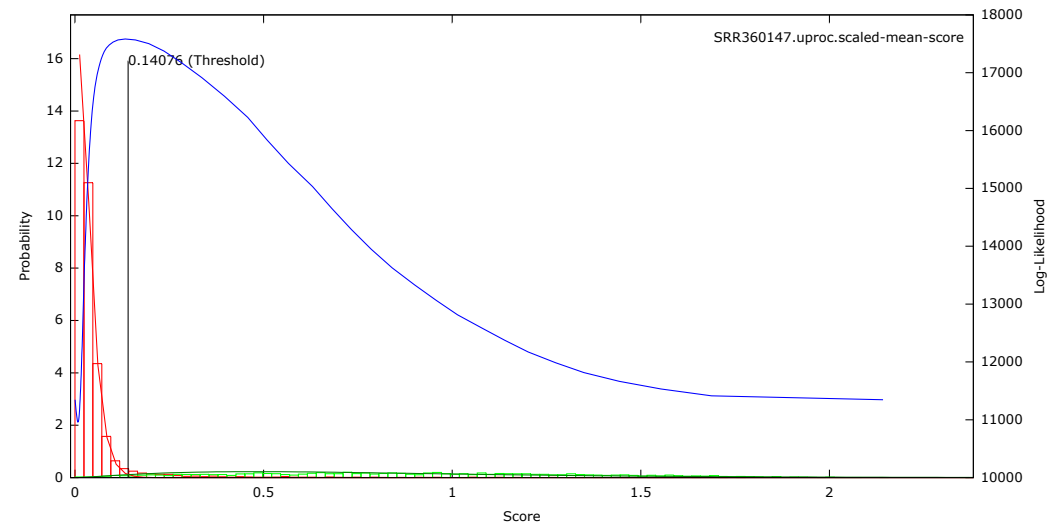

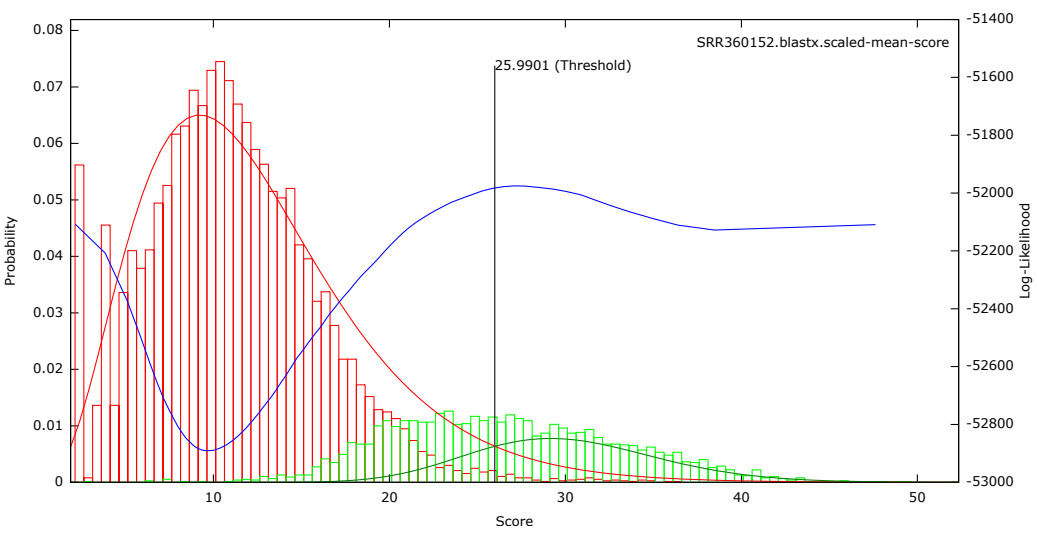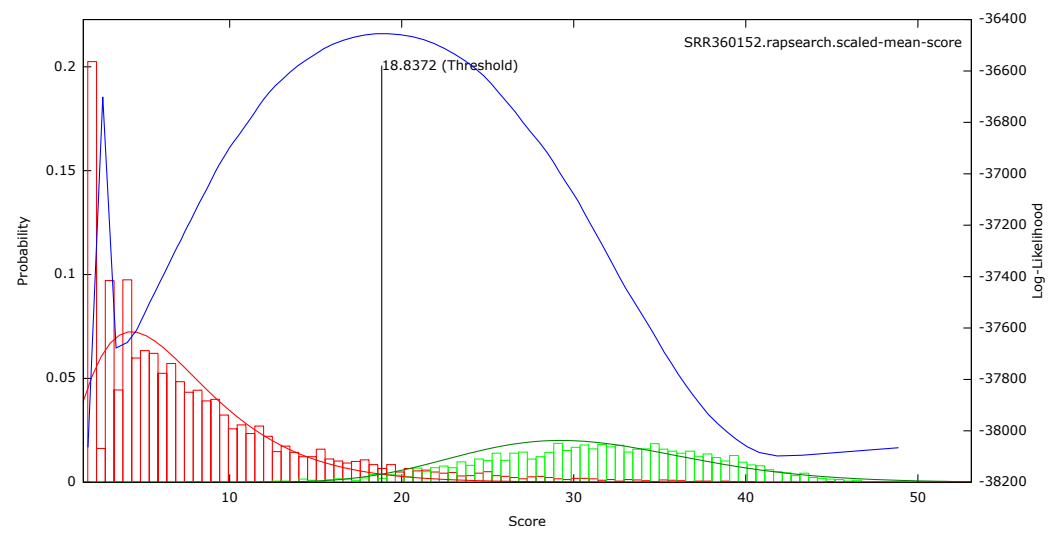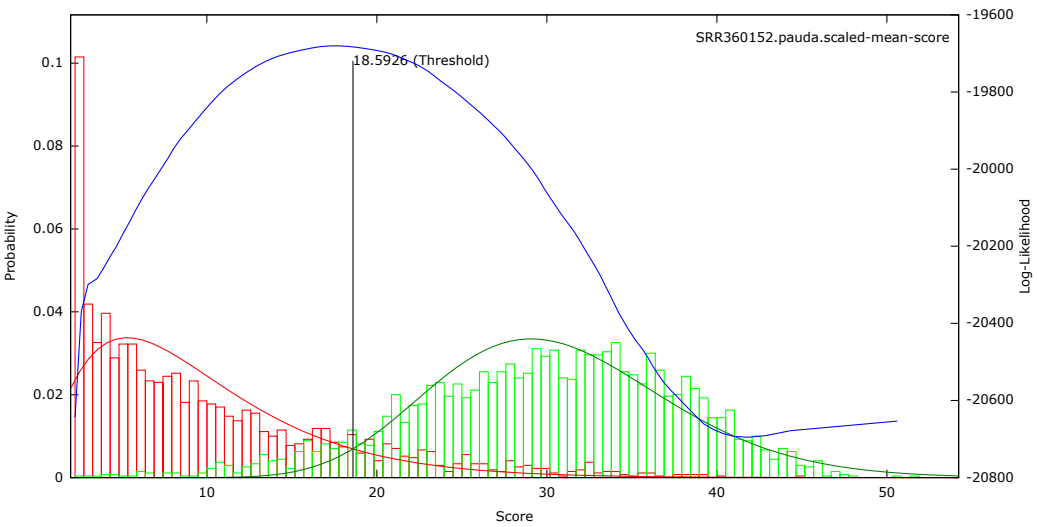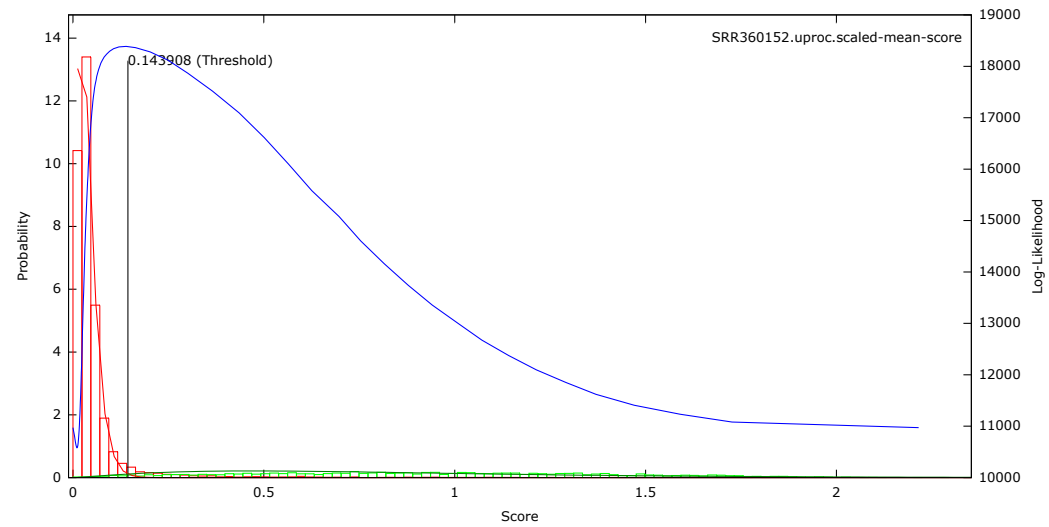

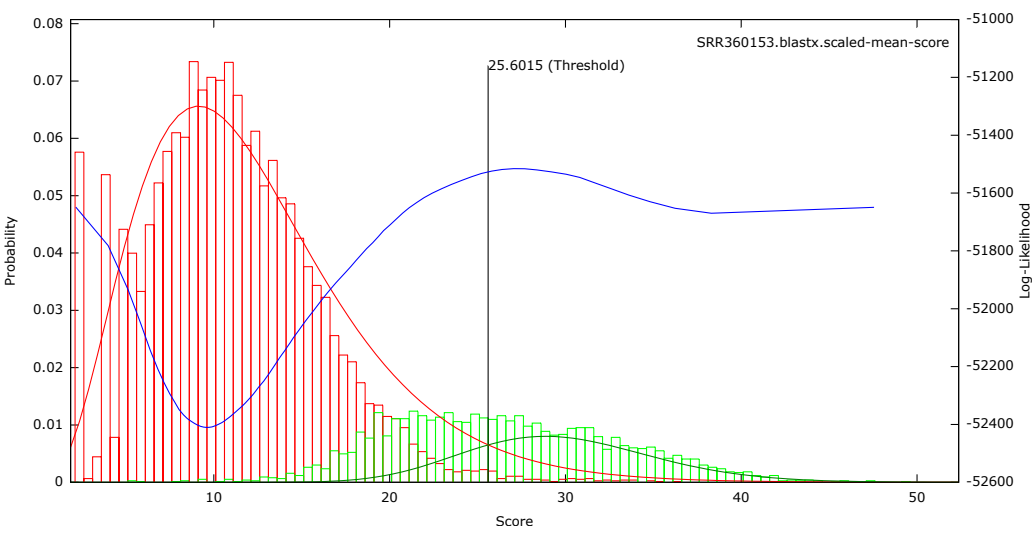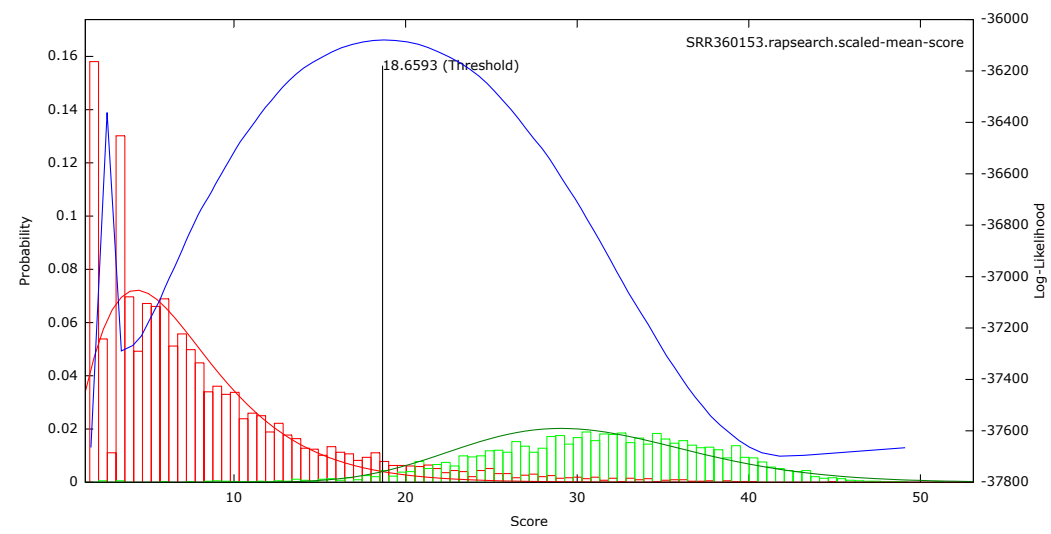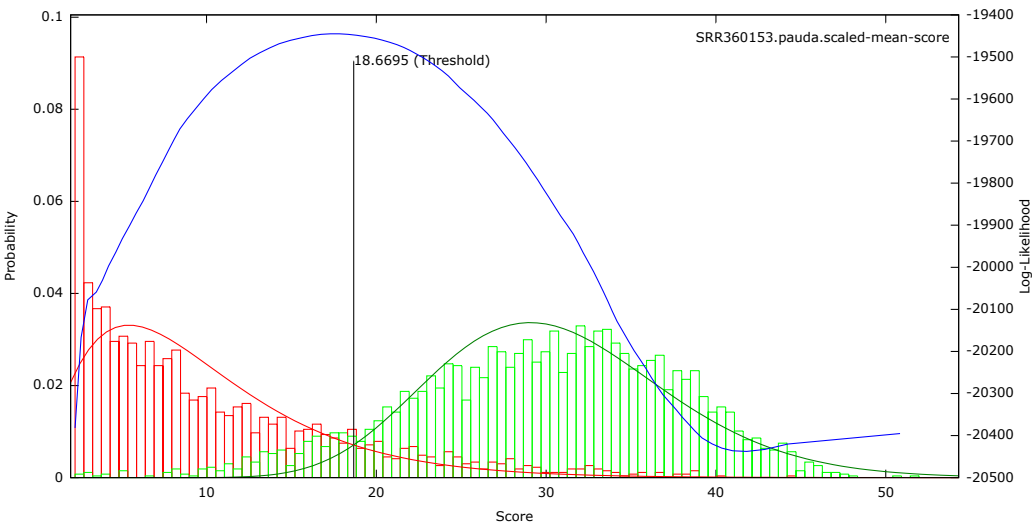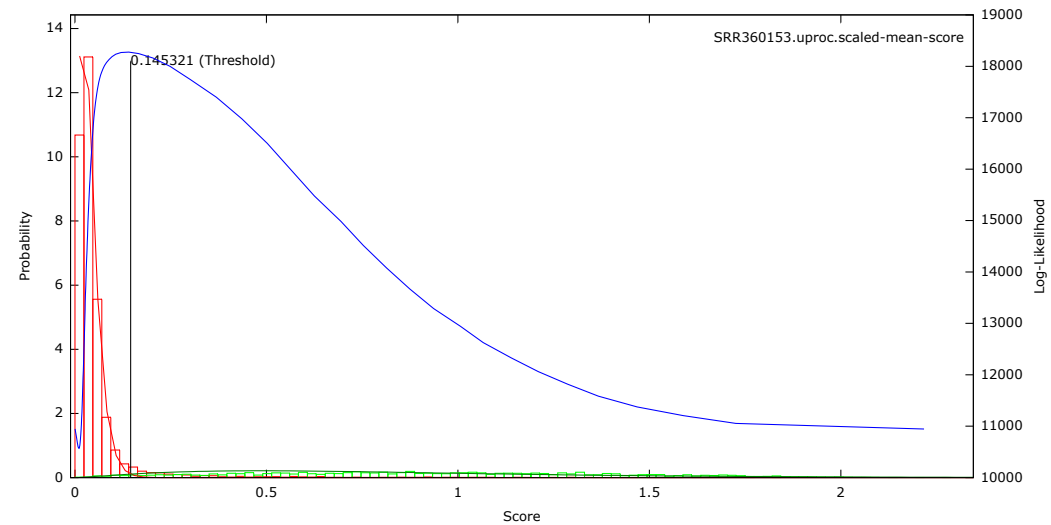

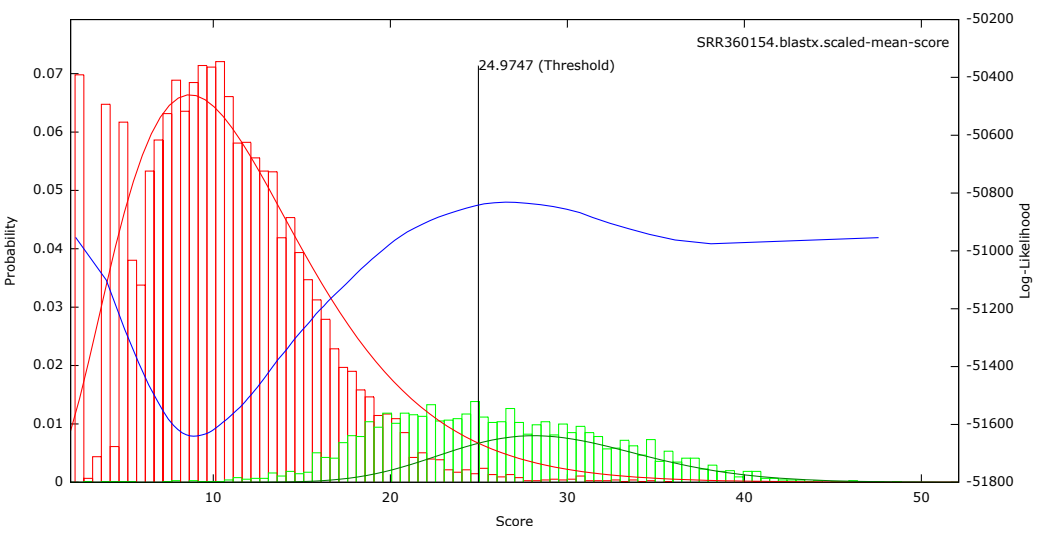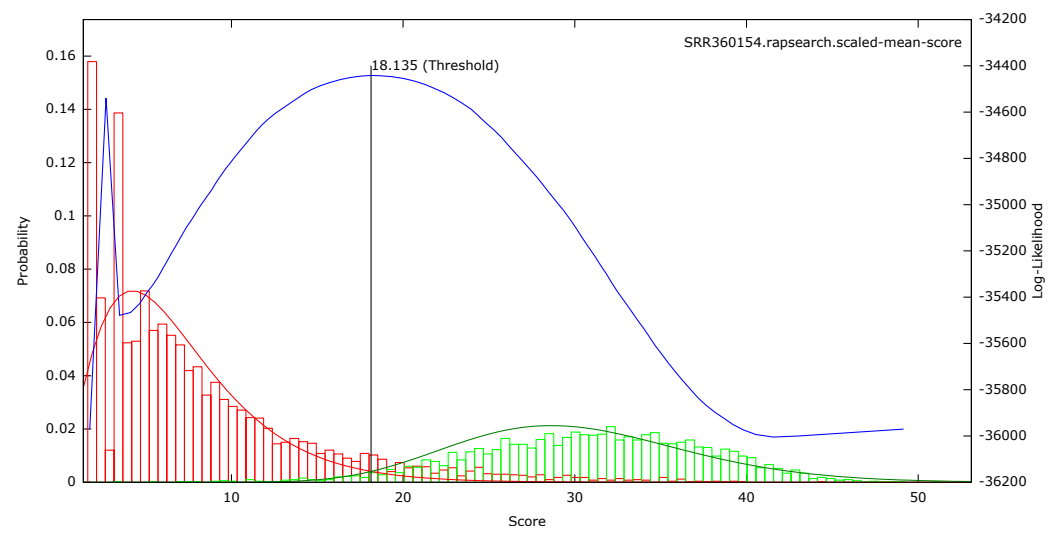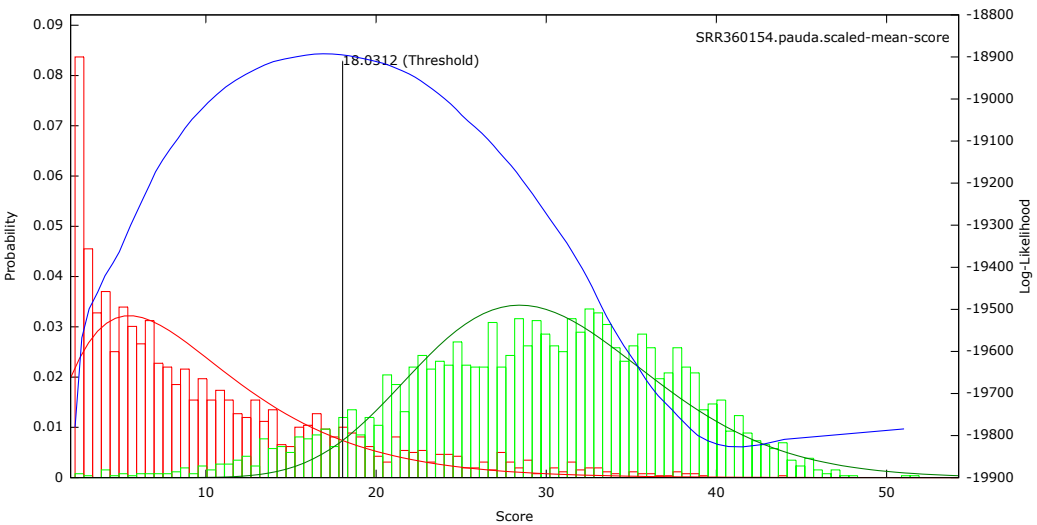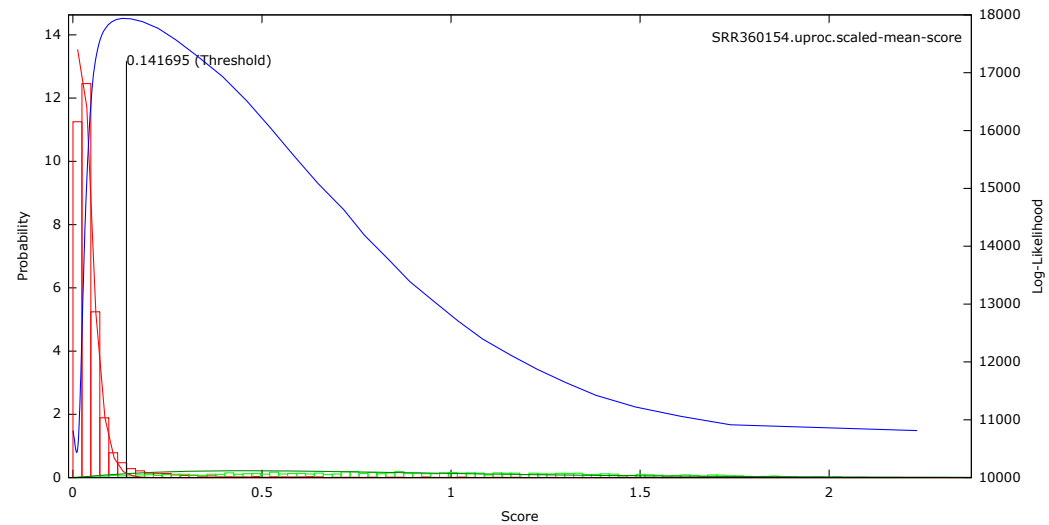

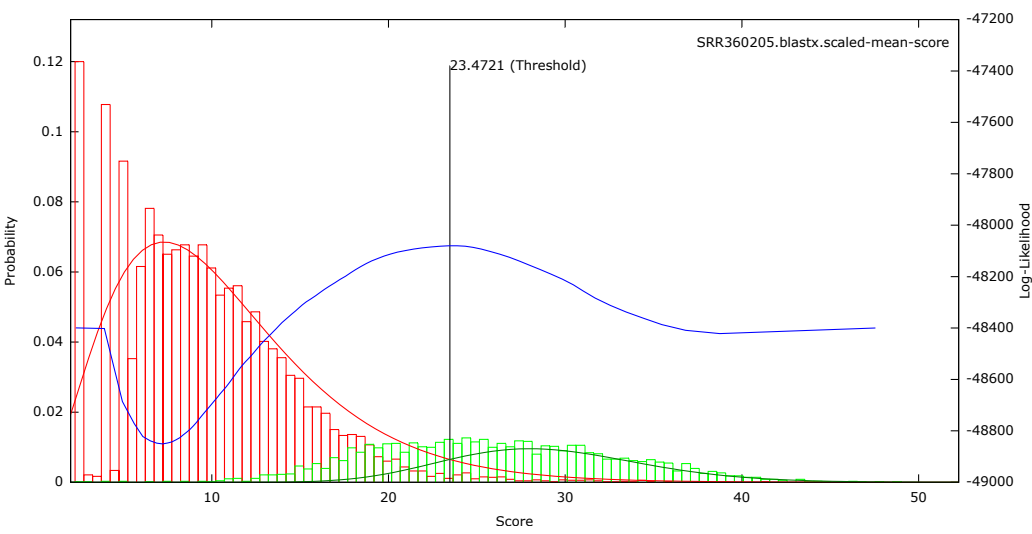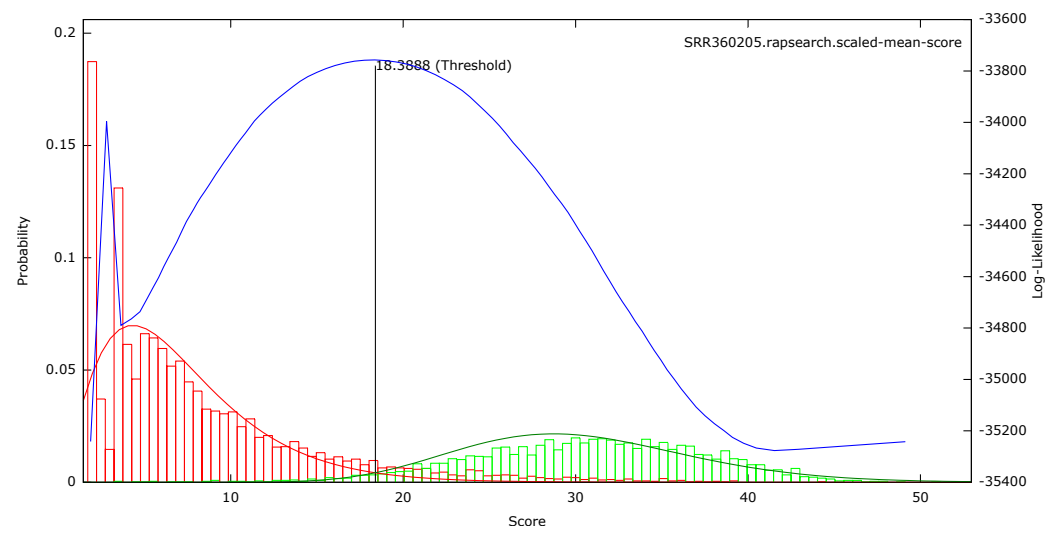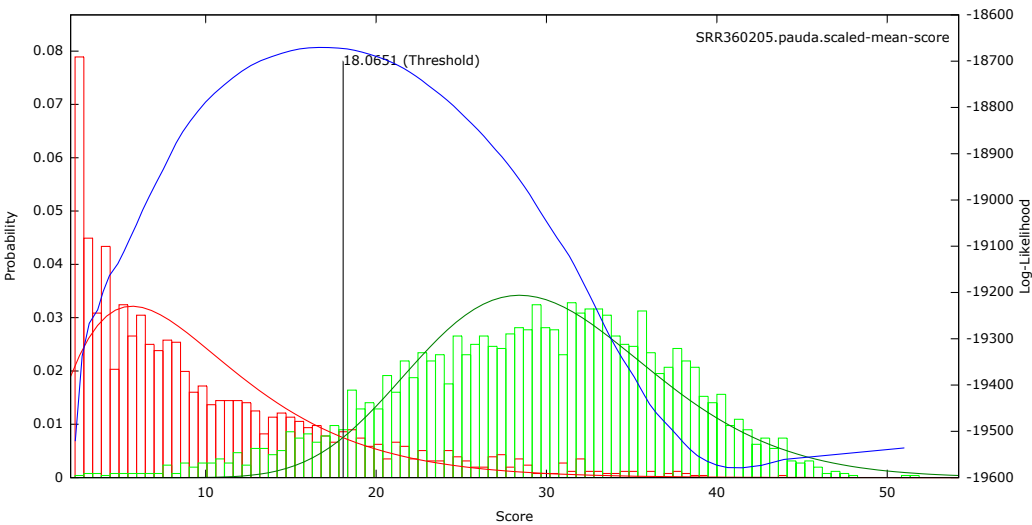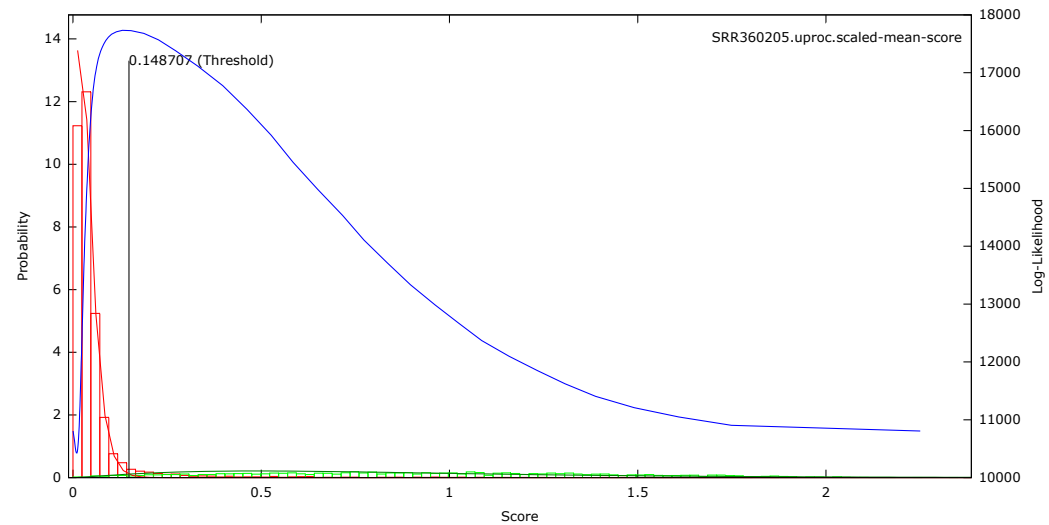

Supplement: Supplementary file 5 — Additional file 5:Histograms of scores. The distributions of the mean–score evidence measures are modeled by two Gaussian distributions and fitted by an unsupervised Expectation–Maximization algorithm. The scaled mean–score evidence values are modeled by two Gamma distributions. The evidence value histograms of the falsely predicted and the annotated functions are colored in red and green, respectively. The curves correspond to the probability distributions of the two component mixture model. Although the probability density curves are shown colored in the plot, the fitting of the model was performed in an unsupervised manner. Histograms were generated for all combinations of samples and tools. Even though the algorithms have converged to the maximum likelihood solution, the resulting models sometimes do not fit the observed data very well. (PDF 546 KB) [file 12864_2014_6719_MOESM5_ESM.pdf]

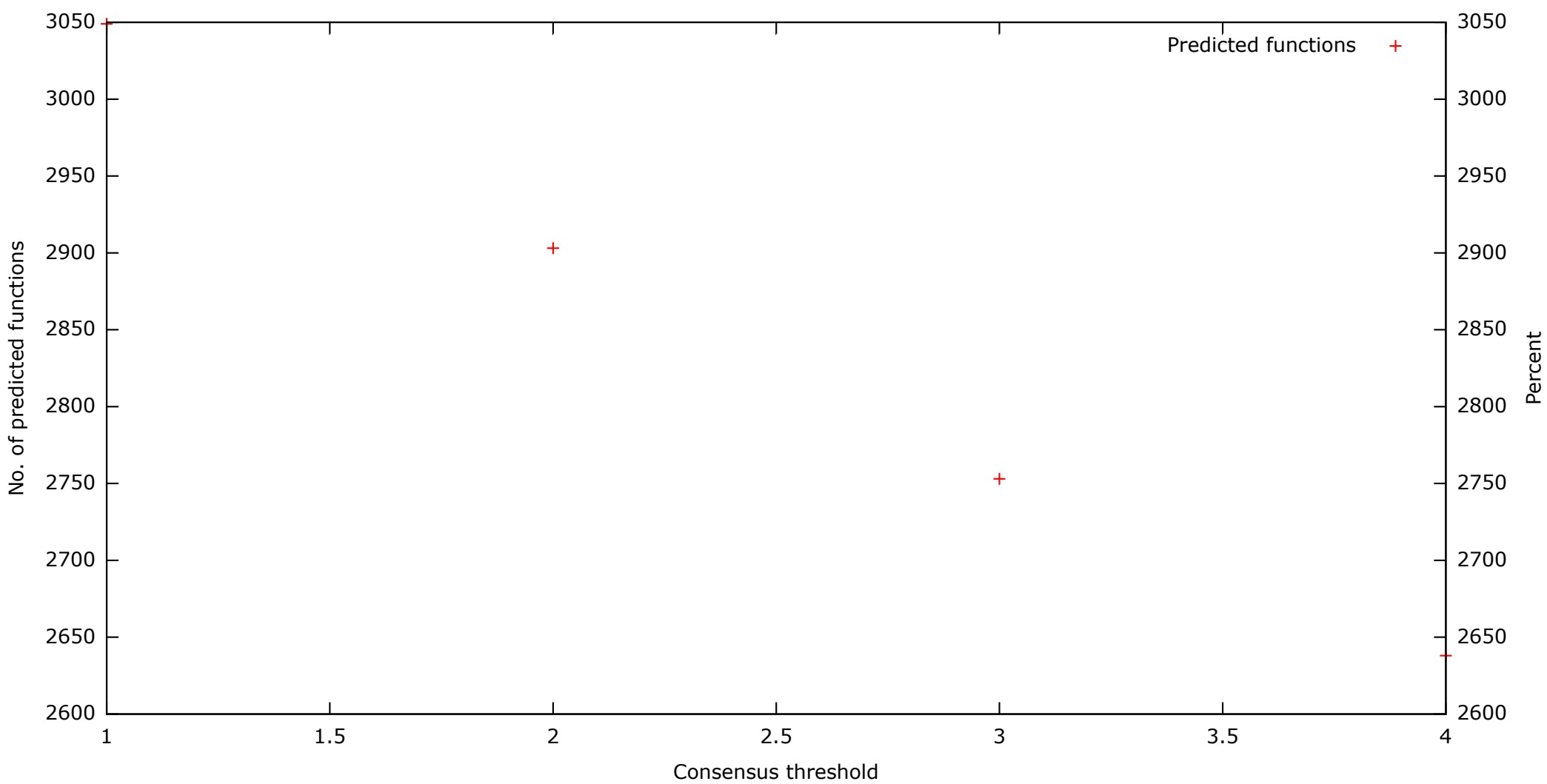

Supplement: Supplementary file 9 — Additional file 9:Prediction on V. dahliae JR2. The scores and predictions calculated for V. dahliae JR2 using the full KEGG database. (ZIP 399 KB) [file 12864_2014_6719_MOESM9_ESM.zip › consensus.histogram.pdf]

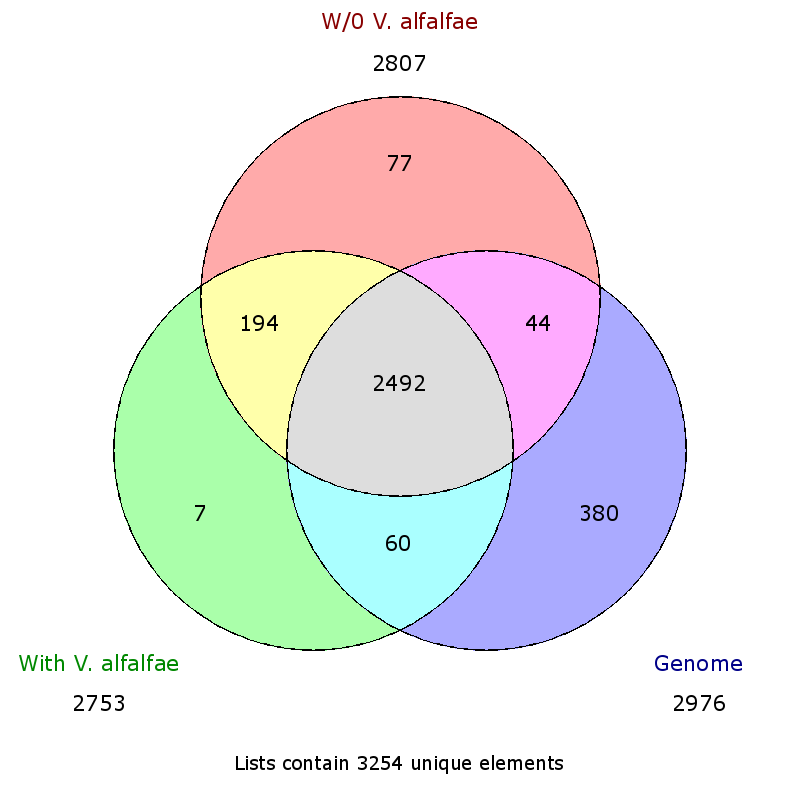

Supplement: Supplementary file 10 — Additional file 10:Comparison of the predicted functions of V. dahliae JR2. Venn diagram comparing the number of predicted functions from the transcriptome using the full database, after removing V. alfalfae and the genome, respectively. (PNG 26 KB) [file 12864_2014_6719_MOESM10_ESM.png]
