# Supplementary material for: Predicting the functional repertoire of an organism from unassembled RNA–seq data
Source: BMC Genomics. 2014 Nov 20;15(1):1003. doi: 10.1186/1471-2164-15-1003 (PMC4258056; doi:10.1186/1471-2164-15-1003)
Supplement: Supplementary file 2 — Additional file 2:Mapping statistics. For each tool and sample, the number of mapped reads, hits, and distinct functions are given. PAUDA only maps approximately 17% of the query reads to the amino acid sequences in the database while BLASTX and RAPSearch both map about 40% of them. Most reads were classified by UProC. Because no stringent threshold is used for the mapping, all tools hit many false functions (about 3000 true functions are expected). Therefore, a filtering for false positive hits is required. (PDF 30 KB) [file 12864_2014_6719_MOESM2_ESM.pdf]

	SRR360147	SRR360152	SRR360153	SRR360154	SRR360205
Reads	9,440,081	29,563,499	27,732,601	22,052,435	20,152,768
**BLASTX** (on 10 percent sub–sample)					
Reads	673,810	2,201,408	2,062,115	1,638,042	1,423,378
Hits	59,075,770	198,272,791	185,708,105	145,265,924	134,225,556
Functions	14,912	15,898	15,845	15,749	15,256
**RAPSearch**					
Reads	3,333,121	11,035,176	10,292,597	8,089,149	7,368,753
Hits	514,834,565	1,725,976,743	1,613,889,721	1,263,534,598	1,150,088,746
Functions	9,620	12,243	12,076	11,694	11,550
**PAUDA**					
Reads	1,230,406	4,097,430	3,832,981	3,002,024	2,728,938
Hits	6,581,398	22,162,018	20,807,543	16,315,218	14,833,824
Functions	4,983	6,297	6,201	5,970	5,944
**UProC**					
Reads	5,251,334	17,169,897	16,118,692	12,675,014	11,534,621
Hits	5,251,334	17,169,897	16,118,692	12,675,014	11,534,621
Functions	15,214	15,792	15,745	15,646	15,645
