# Supplementary material for: Predicting the functional repertoire of an organism from unassembled RNA–seq data
Source: BMC Genomics. 2014 Nov 20;15(1):1003. doi: 10.1186/1471-2164-15-1003 (PMC4258056; doi:10.1186/1471-2164-15-1003)
Supplement: Supplementary file 6 — Additional file 6:Performance for Gaussian model–based filtering on mean–score values. Quality of the filtering for all tools regarding the mean–scores. True–Positive–Rate (TPR), False–Positive–Rate (FPR), Precision (Positive–Predictive–Values) and F1–Score (F1) were calculated after filtering the single samples. (PDF 28 KB) [file 12864_2014_6719_MOESM6_ESM.pdf]

**BLASTX**	TPR	FPR	Precision	F1
SRR360147	0.916084	0.0812977	0.733894	0.814930
SRR360152	0.929116	0.0834760	0.731481	0.818538
SRR360153	0.932613	0.0886105	0.720354	0.812855
SRR360154	0.925620	0.0819200	0.734426	0.819013
SRR360205	0.938970	0.0931228	0.711636	0.809648
Average	0.928481	0.0856854	0.726358	0.814997

**RAPSearch**	TPR	FPR	Precision	F1
SRR360147	0.966624	0.145558	0.619096	0.754778
SRR360152	0.966624	0.179166	0.569049	0.716372
SRR360153	0.966942	0.177999	0.570732	0.717791
SRR360154	0.965671	0.176599	0.572344	0.718713
SRR360205	0.967260	0.180100	0.567936	0.715663
Average	0.966624	0.171884	0.579831	0.724663

**PAUDA**	TPR	FPR	Precision	F1
SRR360147	0.938970	0.059592	0.794086	0.860472
SRR360152	0.952003	0.105804	0.687715	0.798560
SRR360153	0.948824	0.102536	0.693702	0.801450
SRR360154	0.944056	0.097946	0.702294	0.805424
SRR360205	0.950095	0.103236	0.692539	0.801126
Average	0.946790	0.093823	0.714067	0.813406

**UProC**	TPR	FPR	Precision	F1
SRR360147	0.947552	0.086666	0.727961	0.823367
SRR360152	0.951049	0.096390	0.707161	0.811170
SRR360153	0.951685	0.094834	0.710657	0.813698
SRR360154	0.949460	0.095457	0.708828	0.811685
SRR360205	0.948824	0.093823	0.712241	0.813684
Average	0.949714	0.093434	0.713370	0.814721
