# Supplementary material for: Predicting the functional repertoire of an organism from unassembled RNA–seq data
Source: BMC Genomics. 2014 Nov 20;15(1):1003. doi: 10.1186/1471-2164-15-1003 (PMC4258056; doi:10.1186/1471-2164-15-1003)
Supplement: Supplementary file 7 — Additional file 7:Performance for Gamma model–based filtering on scaled mean–score values. Quality of the filtering for all tools regarding the scaled mean–scores. True–Positive–Rate (TPR), False–Positive–Rate (FPR), Precision (Positive–Predictive–Values) and F1–Score (F1) were calculated after filtering the single samples. (PDF 28 KB) [file 12864_2014_6719_MOESM7_ESM.pdf]

**BLASTX**	TPR	FPR	Precision	F1
SRR360147	0.711380	0.021472	0.890215	0.790813
SRR360152	0.509536	0.006846	0.947960	0.662808
SRR360153	0.445327	0.004979	0.956314	0.607677
SRR360154	0.459313	0.005290	0.955056	0.620305
SRR360205	0.621424	0.011358	0.930509	0.745188
Average	0.549396	0.009989	0.936011	0.685358

**RAPSearch**	TPR	FPR	Precision	F1
SRR360147	0.939288	0.044189	0.838774	0.886190
SRR360152	0.938652	0.043099	0.842030	0.887720
SRR360153	0.936109	0.041310	0.847238	0.889459
SRR360154	0.938652	0.043255	0.841550	0.887453
SRR360205	0.934838	0.041077	0.847795	0.889191
Average	0.937508	0.042586	0.843477	0.888003

**PAUDA**	TPR	FPR	Precision	F1
SRR360147	0.845518	0.018982	0.915978	0.879339
SRR360152	0.890019	0.026295	0.892288	0.891152
SRR360153	0.888112	0.026295	0.892082	0.890092
SRR360154	0.866497	0.021628	0.907457	0.886504
SRR360205	0.875079	0.023106	0.902623	0.888638
Average	0.873045	0.023261	0.902086	0.887145

**UProC**	TPR	FPR	Precision	F1
SRR360147	0.938334	0.042633	0.843429	0.888354
SRR360152	0.940877	0.049012	0.824513	0.878860
SRR360153	0.942149	0.050568	0.820144	0.876923
SRR360154	0.941513	0.048934	0.824840	0.879323
SRR360205	0.938334	0.046756	0.830847	0.881326
Average	0.940241	0.047581	0.828755	0.880957
