# Supplementary material for: Predicting the functional repertoire of an organism from unassembled RNA–seq data
Source: BMC Genomics. 2014 Nov 20;15(1):1003. doi: 10.1186/1471-2164-15-1003 (PMC4258056; doi:10.1186/1471-2164-15-1003)
Supplement: Supplementary file 8 — Additional file 8:Performance after filtering and different consensus thresholds. To combine the samples, different consensus thresholds c were applied. A function was predicted present if contained in at least c samples after filtering the samples. See also Figures2 and3. (PDF 32 KB) [file 12864_2014_6719_MOESM8_ESM.pdf]

Mean-Score

Consensus	BLASTX FPR	BLASTX TPR	BLASTX F1	BLASTX FP	BLASTX TP	RAPSearch FPR	RAPSearch TPR	RAPSearch F1	RAPSearch FP	RAPSearch TP
1	0.1577	0.9456	0.7302	2027	2975	0.2833	0.9692	0.6200	3641	3049
2	0.0877	0.9380	0.8170	1127	2951	0.1976	0.9673	0.6972	2540	3043
3	0.0720	0.9339	0.8384	925	2938	0.1519	0.9666	0.7473	1952	3041
4	0.0616	0.9228	0.8487	792	2903	0.1261	0.9660	0.7786	1621	3039
5	0.0495	0.9021	0.8574	636	2838	0.1006	0.9641	0.8118	1293	3033

	PAUDA FPR	PAUDA TPR	PAUDA F1	PAUDA FP	PAUDA TP	UProC FPR	UProC TPR	UProC F1	UProC FP	UProC TP
1	0.1808	0.9568	0.7099	2324	3010	0.1328	0.9542	0.7644	1707	3002
2	0.1158	0.9542	0.7863	1488	3002	0.0998	0.9523	0.8070	1283	2996
3	0.0771	0.9485	0.8381	991	2984	0.0875	0.9504	0.8236	1125	2990
4	0.0559	0.9425	0.8682	719	2965	0.0787	0.9479	0.8353	1012	2982
5	0.0395	0.9320	0.8904	508	2932	0.0683	0.9437	0.8491	878	2969

Scaled-Mean-Score

Consensus	BLASTX FPR	BLASTX TPR	BLASTX F1	BLASTX FP	BLASTX TP	RAPSearch FPR	RAPSearch TPR	RAPSearch F1	RAPSearch FP	RAPSearch TP
1	0.0318	0.7645	0.8070	409	2405	0.0448	0.9428	0.8870	576	2966
2	0.0107	0.6122	0.7395	137	1926	0.0421	0.9380	0.8891	541	2951
3	0.0089	0.5620	0.7031	115	1768	0.0407	0.9348	0.8899	523	2941
4	0.0055	0.4809	0.6397	71	1513	0.0398	0.9329	0.8903	512	2935
5	0.0052	0.4558	0.6172	67	1434	0.0367	0.9275	0.8929	472	2918

	PAUDA FPR	PAUDA TPR	PAUDA F1	PAUDA FP	PAUDA TP	UProC FPR	UProC TPR	UProC F1	UProC FP	UProC TP
1	0.0261	0.8913	0.8921	336	2804	0.0547	0.9450	0.8716	703	2973
2	0.0244	0.8802	0.8892	313	2769	0.0511	0.9425	0.8762	657	2965
3	0.0226	0.8738	0.8888	291	2749	0.0482	0.9412	0.8803	620	2961
4	0.0208	0.8624	0.8857	267	2713	0.0454	0.9374	0.8832	583	2949
5	0.0159	0.8141	0.8664	205	2561	0.0396	0.9358	0.8923	509	2944
